# A Mouse Model with a Frameshift Mutation in the Nuclear Factor I/X ( *NFIX* ) Gene Has Phenotypic Features of Marshall‐Smith Syndrome

**DOI:** 10.1002/jbm4.10739

**Published:** 2023-03-30

**Authors:** Kreepa G. Kooblall, Mark Stevenson, Michelle Stewart, Lachlan Harris, Oressia Zalucki, Hannah Dewhurst, Natalie Butterfield, Houfu Leng, Tertius A. Hough, Da Ma, Bernard Siow, Paul Potter, Roger D. Cox, Stephen D.M. Brown, Nicole Horwood, Benjamin Wright, Helen Lockstone, David Buck, Tonia L. Vincent, Fadil M. Hannan, J.H. Duncan Bassett, Graham R. Williams, Kate E. Lines, Michael Piper, Sara Wells, Lydia Teboul, Raoul C. Hennekam, Rajesh V. Thakker

**Affiliations:** ^1^ Academic Endocrine Unit, Radcliffe Department of Medicine, Oxford Centre for Diabetes, Endocrinology and Metabolism (OCDEM) University of Oxford Oxford UK; ^2^ MRC Harwell, Mary Lyon Centre Harwell Science and Innovation Campus Oxfordshire UK; ^3^ The Francis Crick Institute London UK; ^4^ The School of Biomedical Sciences and The Queensland Brain Institute The University of Queensland Brisbane Australia; ^5^ Molecular Endocrinology Laboratory, Department of Metabolism, Digestion and Reproduction, Imperial College London Hammersmith Hospital London UK; ^6^ Centre for OA Pathogenesis Versus Arthritis, The Kennedy Institute of Rheumatology, Nuffield Department of Orthopaedics, Rheumatology and Musculoskeletal Sciences (NDORMS) Medical Sciences Division University of Oxford Oxford UK; ^7^ Department of Internal Medicine Wake Forest University School of Medicine Winston‐Salem NC USA; ^8^ Oxford Genomics Centre, The Wellcome Centre for Human Genetics University of Oxford Oxford UK; ^9^ Nuffield Department of Women's and Reproductive Health University of Oxford Oxford UK; ^10^ Department of Pediatrics, Amsterdam UMC University of Amsterdam Amsterdam The Netherlands

**Keywords:** NFIX, kyphosis, osteopenia, brain abnormalities, frameshift mutation

## Abstract

The nuclear factor I/X (*NFIX*) gene encodes a ubiquitously expressed transcription factor whose mutations lead to two allelic disorders characterized by developmental, skeletal, and neural abnormalities, namely, Malan syndrome (MAL) and Marshall–Smith syndrome (MSS). *NFIX* mutations associated with MAL mainly cluster in exon 2 and are cleared by nonsense‐mediated decay (NMD) leading to NFIX haploinsufficiency, whereas *NFIX* mutations associated with MSS are clustered in exons 6–10 and escape NMD and result in the production of dominant‐negative mutant NFIX proteins. Thus, different *NFIX* mutations have distinct consequences on *NFIX* expression. To elucidate the *in vivo* effects of MSS‐associated *NFIX* exon 7 mutations, we used CRISPR‐Cas9 to generate mouse models with exon 7 deletions that comprised: a frameshift deletion of two nucleotides (*Nfix* Del2); in‐frame deletion of 24 nucleotides (*Nfix* Del24); and deletion of 140 nucleotides (*Nfix* Del140). *Nfix* ^*+/Del2*^, *Nfix* ^*+/Del24*^, *Nfix* ^*+/Del140*^, *Nfix* ^*Del24/Del24*^, and *Nfix* ^*Del140/Del140*^ mice were viable, normal, and fertile, with no skeletal abnormalities, but *Nfix* ^*Del2/Del2*^ mice had significantly reduced viability (*p* < 0.002) and died at 2–3 weeks of age. *Nfix* Del2 was not cleared by NMD, and *Nfix^Del2/Del2^* mice, when compared to *Nfix* ^*+/+*^ and *Nfix* ^*+/Del2*^ mice, had: growth retardation; short stature with kyphosis; reduced skull length; marked porosity of the vertebrae with decreased vertebral and femoral bone mineral content; and reduced caudal vertebrae height and femur length. Plasma biochemistry analysis revealed *Nfix* ^*Del2/Del2*^ mice to have increased total alkaline phosphatase activity but decreased C‐terminal telopeptide and procollagen‐type‐1‐N‐terminal propeptide concentrations compared to *Nfix* ^*+/+*^ and *Nfix* ^*+/Del2*^ mice. *Nfix* ^*Del2/Del2*^ mice were also found to have enlarged cerebral cortices and ventricular areas but smaller dentate gyrus compared to *Nfix* ^*+/+*^ mice. Thus, *Nfix* ^*Del2/Del2*^ mice provide a model for studying the *in vivo* effects of NFIX mutants that escape NMD and result in developmental abnormalities of the skeletal and neural tissues that are associated with MSS. © 2023 The Authors. *JBMR Plus* published by Wiley Periodicals LLC on behalf of American Society for Bone and Mineral Research.

## Graphical abstract

A mouse model for Marshall–Smith syndrome.

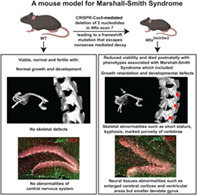

## Introduction

The nuclear factor I/X (*NFIX*) gene (MIM #164005),^(^ ^1^, ^2^, ^3^ ^)^ located on chromosome 19p13.2,^(^ ^4^ ^)^ consists of 11 exons (Fig. S1) that encode 14 transcripts, of which 11 are protein coding. *NFIX* encodes a ubiquitously expressed transcription factor that forms part of the *NFI* gene family, which in mammals consists of *NFIA*, *NFIB*, *NFIC*, and *NFIX*. These transcription factors share a highly conserved N‐terminal DNA binding and dimerization domain, which bind as homo‐ or heterodimers to the consensus palindromic sequence 5′‐TTGGC(N5)GCCAA‐3′ present in the promoter regions of viral and cellular genes,^(^ ^5^ ^)^ and a variable C‐terminal transactivation/repression domain, which can potentially provide a range of preferential interactions with other proteins to either activate or suppress transcription.^(^ ^6^, ^7^, ^8^, ^9^ ^)^ NFI transcription factors play important roles in the regulation of stem cell differentiation, quiescence, and differentiation during the development of organs that include lung, kidney, liver, blood, heart, skeleton, and the nervous system.^(^ ^6^, ^10^ ^)^

Heterozygous mutations in the *NFIX* gene can lead to two rare allelic disorders, Malan syndrome (MAL; MIM #614753) and Marshall‐Smith syndrome (MSS; MIM #602535).^(^ ^1^, ^9^ ^)^ MAL is an overgrowth disorder, characterized by a slender habitus, long hands and advanced bone age, moderate to severe intellectual disability, unusual facial phenotype consisting of a long, triangular face with a prominent forehead, everted lower lip and prominent chin, and behavioral problems, which are usually dominated by anxieties and, less frequently, by aggression^(^ ^9^, ^10^, ^11^, ^12^ ^)^ (Table S1). The missense, nonsense, and frameshift *NFIX* variants reported in MAL patients predominantly affect exon 2 (Fig. S1), which encodes the highly conserved N‐terminal DNA binding and dimerization domain of the NFIX protein.^(^ ^1^, ^3^, ^13^, ^14^ ^)^ Entire gene deletions and *NFIX* mutations observed in MAL patients are predicted to be cleared by nonsense‐mediated mRNA decay (NMD) and lead to NFIX haploinsufficiency.^(^ ^1^, ^2^, ^3^, ^11^, ^13^, ^14^ ^)^ MSS is characterized by short stature with skeletal abnormalities that may include kyphoscoliosis, abnormal bone maturation, craniofacial defects, and osteopenia and be associated with delays in motor and neural development that lead to moderate to severe mental retardation, limited or absent speech, and postnatal failure to thrive.^(^ ^15^, ^16^ ^)^ In addition, MSS patients may have distinctive facial features that include a high forehead, proptosis, blue sclera, anteverted nares, small and retracted mandible, gingival hypertrophy, and hypertrichosis (Table S1). MSS patients may also suffer from respiratory difficulties with upper‐airway obstruction and apneas. The *de novo* frameshift *NFIX* mutations reported in MSS patients are all clustered in exons 6–10 of the *NFIX* gene, which encode the variable C‐terminal transactivation/repression domain (Fig. S1). The different mutations result in the production of aberrant transcripts that escape NMD and lead to the production of dysfunctional truncated NFIX proteins, which are predicted to behave in a dominant‐negative manner.^(^ ^1^, ^2^, ^3^ ^)^ Thus, mutations that affect different regions of the *NFIX* gene have distinct consequences on the resulting transcripts and encoded proteins.

To date, only the *in vivo* consequences of *Nfix* exon 2 deletion, which encodes the conserved N‐terminal DNA binding and dimerization domain, have been studied in mouse models.^(^ ^17^, ^18^ ^)^ In one study wherein *Nfix* exon 2 was replaced with an in‐frame *lacZ* reporter gene, *Nfix* ^*+/lacZ*^ mice were reported to have normal survival, but reduced body weight, while *Nfix* ^*lacZ/lacZ*^ mice developed skeletal abnormalities due to defects in ossification that resulted in kyphosis and neurological abnormalities such as partial agenesis of the corpus callosum that was associated with hydrocephalus.^(^ ^17^ ^)^ In other studies wherein an *Nfix* null allele was initially generated via Cre‐recombinase‐mediated excision of *Nfix* exon 2, the heterozygous *Nfix* ^*+/−*^ mice also had normal survival but with neurological abnormalities,^(^ ^19^ ^)^ and the homozygous *Nfix* ^*−/−*^ mice had neurological defects that included dysgenesis of the corpus callosum but did not have skeletal abnormalities.^(^ ^18^ ^)^ Moreover, *Nfix* ^*−/−*^ mice are reported to have severe delay in intermediate progenitor cells during forebrain development^(^ ^20^ ^)^ and smaller muscle fibers with impairment of muscle regeneration despite the lack of skeletal defects^(^ ^21^ ^)^ (Table S1). These *Nfix*‐deficient mice with targeted deletions of exon 2 are reported to be representative of MAL. Therefore, to establish potential representative models for MSS, we generated *Nfix* mouse models with frameshift mutations in exon 7, which is the most commonly mutated exon in MSS patients.^(^ ^1^, ^2^, ^3^ ^)^

## Materials and Methods

### Study approval

All animal studies were approved by the Medical Research Council Harwell Institute Ethical Review Committee and were licensed under the Animal (Scientific Procedures) Act 1986, issued by the UK Government Home Office Department (PPL30/2433 and PPL30/3271).

### Generation of mutant mice and genotyping analysis

Mice were generated using the CRISPR/Cas9 system,^(^ ^22^ ^)^ and genotyping was performed by PCR amplification using genomic DNA and confirmed by RT‐PCR using total RNA extracted, as described in Data S2 Materials and Methods.

### Cell lines and in vitro expression assays

Murine embryonic fibroblast (MEF) cells and monkey kidney fibroblast (COS‐7) cells that were used for RNA sequencing analysis or transiently transfected with wild‐type (WT) or mutant murine *Nfix* cDNA expression constructs and luciferase reporter constructs were utilized for qRT‐PCR, Western blot, and immunofluorescence analyses, as detailed in Data S2 Materials and Methods.

### Phenotype analysis

Blood samples were collected and used for plasma biochemical analysis,^(^ ^22^ ^)^ and skeletons and tissues of WT and mutant mice were prepared and used for imaging and histological analyses, as detailed in Data S2 Materials and Methods.^(^ ^23^ ^)^

### Statistical analysis

Data are expressed as mean and standard deviation (SD) or standard errors of mean (SEM). All analyses were performed using Prism (GraphPad), and a value of *p* < 0.05 was considered significant for all analyses as described in Data S2 Materials and Methods.

## Results

### Establishment of mutant *Nfix* mouse models with targeted mutations of exon 7

To derive mouse models with frameshift mutations that affect the variable C‐terminal transactivation or repression domain of the *NFIX* gene, the CRISPR‐Cas9 system was used to target exon 7 of the murine *Nfix* gene. Following injection of Cas9 mRNA and *Nfix* guide RNA into C57BL/6J embryos, founder mice were generated from which three mutant *Nfix* lines comprising deletions of two nucleotides (Del2), 24 nucleotides (Del24), and 140 nucleotides (Del140) were established. More specifically, *Nfix* Del2 consists of a frameshift two‐nucleotide deletion from position +49,580 to +49,581 relative to the translation start site (TSS), *Nfix* Del24 contains an in‐frame 24‐nucleotide deletion (from position +49,561 to +49,584 relative to the TSS), and *Nfix* Del140 contains a 140‐nucleotide deletion (from position +49,577 to +49,716 relative to the TSS) and comprised 53 nucleotides from exon 7 and 87 nucleotides of intron 7 (Fig. S2*A*).

Heterozygous *Nfix* mice (*Nfix* ^*+/Del2*^, *Nfix* ^*+/Del24*^, and *Nfix* ^*+/Del140*^) were viable and intercrossed within each line to generate WT (*Nfix* ^*+/+*^), heterozygous (*Nfix* ^*+/Del2*^, *Nfix* ^*+/Del24*^, and *Nfix* ^*+/Del140*^) and homozygous (*Nfix* ^*Del2/Del2*^, *Nfix* ^*Del24/Del24*^, and *Nfix* ^*Del140/Del140*^) mice. Genotypes were confirmed and validated by PCR amplification of exon 7, Sanger DNA sequencing, and, in the case of *Nfix* Del2 mice, using *Nla*III restriction endonuclease digestion analysis (Fig. S2 *B*–*D*). *Nfix* ^*+/Del2*^, *Nfix* ^*+/Del24*^, *Nfix* ^*Del24/Del24*^, *Nfix* ^*+/Del140*^, and *Nfix* ^*Del140/Del140*^ mice were viable, normal, and fertile, but *Nfix* ^*Del2/Del2*^ mice were subviable by 21 days post term (P21) due to early death around 2–3 weeks of age (*p* = 0.002; Table 1). Thus, deviation from the normal Mendelian ratio (1:2:1) was not observed in the *Nfix* ^*Del140/Del140*^ and *Nfix* ^*Del24/Del24*^ mice at E18.5, P21, or 12 weeks and in the *Nfix* ^*Del2/Del2*^ mice at E18.5. However, the *Nfix* ^*Del2/Del2*^ mice deviated significantly (*p* = 0.002) from the expected Mendelian ratio at P21 due to early death at 2–3 weeks, indicative of reduced viability of the *Nfix* ^*Del2/Del2*^ mice. Moreover, the numbers of *Nfix* ^*+/+*^, *Nfix* ^*+/Del2*^, and *Nfix* ^*Del2/Del2*^ mouse embryos at day 18.5 (E18.5) did not deviate from the expected 1:2:1 Mendelian ratio (Table 1), thereby suggesting that the life‐limiting mutational effects in *Nfix* ^*Del2/Del2*^ mice are manifested between E18.5 and P21.

**Table 1:** Mendelian Ratios and Binomial Distribution Analysis of Wild‐Type, *Nfix* Del2, Del24, and Del140 Mice from Intercrosses at E18.5, P21, and 12 Weeks of Age

Stage and mutation	Genotype			Total	*p* ^a^
	*Nfix* ^*+/+*^	*Nfix* ^*+/−*^	*Nfix* ^*−/−*^		
E18.5^b^					
Del2	19 (13.5)^c^	26 (27)	9 (13.5)	54	0.101
Del24	14 (16)	31 (32)	19 (16)	64	0.844
Del140	6 (13.25)	27 (26.5)	20 (13.25)	53	0.987
P21^d^					
Del2	74 (71.5)	162 (143)	50 (71.5)	286	0.002**
Del24	86 (68.25)	122 (136.5)	65 (68.25)	273	0.354
Del140	43 (57.25)	122 (114.5)	64 (57.25)	229	0.865
12 weeks					
Del24	86 (67.75)	120 (135.5)	65 (67.75)	271	0.380
Del140	43 (56.75)	120 (113.5)	64 (56.75)	227	0.882

^a^ *p*: probability observed number of homozygotes is significantly different from the expected number of homozygotes, which is 25% of the total number of mice obtained from heterozygotes intercrosses, derived by binomial distribution analysis; **p < 0.01.

^b^ E18.5: embryonic day E18.5.

^c^ Observed numbers are shown with expected numbers shown in parentheses.

^d^ P21: postnatal day 21.

### Effects of three exon 7 mutations (*Nfix* Del2, *Nfix* Del24, and *Nfix* Del140) on *Nfix* transcription and translation

The differences in viability between the homozygous *Nfix* ^*Del2/Del2*^ mutant mice and the homozygous *Nfix* ^*Del24/Del24*^ and *Nfix* ^*Del140/Del140*^ mutant mice suggested that the *Nfix* allelic variants may have different effects on the expression of this transcription factor. We therefore investigated the effects of these *Nfix* allele variants on the transcription and translation of *Nfix*. Murine *Nfix* contains 11 exons that encode eight transcripts (five of which are protein coding), due to alternative splicing of exons 7 and 9 and the use of different transcription initiation sites (ENSMUSG00000001911.16). Thus, alternative splicing may produce WT *Nfix* transcripts that retain exon 7 (*Nfix* long isoform) or shorter conserved isoforms that lack exon 7 (*Nfix* ΔEx7) (Fig. S3*A*). To study the effects of *Nfix* Del2, *Nfix* Del24, and *Nfix* Del140 deletions on splicing of exon 7, RT‐PCR using primers located in exons 6 and 8 and Sanger sequencing were performed on total RNA obtained from MEFs derived from *Nfix* ^*+/+*^, *Nfix* ^*+/Del2*^, *Nfix* ^*Del2/Del2*^, *Nfix* ^*+/Del24*^, *Nfix* ^*Del24/Del24*^, *Nfix* ^*+/Del140*^, and *Nfix* ^*Del140/Del140*^ mice. This revealed that *Nfix* ^*+/+*^ MEFs had the *Nfix* WT long (317 bp) and short WT ΔEx7 (194 bp) isoforms (Fig. S3 *B*–*D*), but the *Nfix* ^*Del2/Del2*^ and *Nfix* ^*Del24/Del24*^ MEFs had mutant *Nfix* long isoforms of 315 and 293 bp, respectively, and the *Nfix* WT short isoform (ΔEx7 of 194 bp) (Fig. S3 *B*,*C*). *Nfix* ^*+/Del2*^ and *Nfix* ^*+/Del24*^ MEFs were confirmed to express a WT *Nfix* long isoform, a mutant *Nfix* long isoform, and the *Nfix* WT ΔEx7 short isoform (Fig. S3 *B*,*C*). In contrast, *Nfix* ^*Del140/Del140*^ MEFs had only the *Nfix* WT ΔEx7 short isoform, thereby suggesting that the 140‐nucleotide deletion, which comprised 53 nucleotides of the 3′ end of exon 7 along with 87 nucleotides from intron 7 that included the donor splice site, led to exon 7 skipping. Sanger DNA sequence analysis confirmed that the sequence of this *Nfix* short isoform from the *Nfix* ^*Del140/Del140*^ MEFs matched the consensus murine sequence of the *Nfix* WT ΔEx7 short isoforms (Fig. S3 *D*). Therefore, the 140‐nucleotide deletion in the *Nfix* Del140 MEFs led to skipping of exon 7 and alternative splicing of exon 6 to exon 8 due to loss of a donor splice site, resulting in a frameshift and the introduction of a stop codon after 81 amino acids, which corresponded to the WT short NFIX isoforms. However, the two‐nucleotide deletion in *Nfix* Del2 MEFs resulted in a frameshift and the introduction of a premature stop codon after 65 amino acids, and the 24‐nucleotide in‐frame deletion in *Nfix* Del24 MEFs predicted the loss of eight amino acids (QGSSPRMA).

**Fig. 1:**
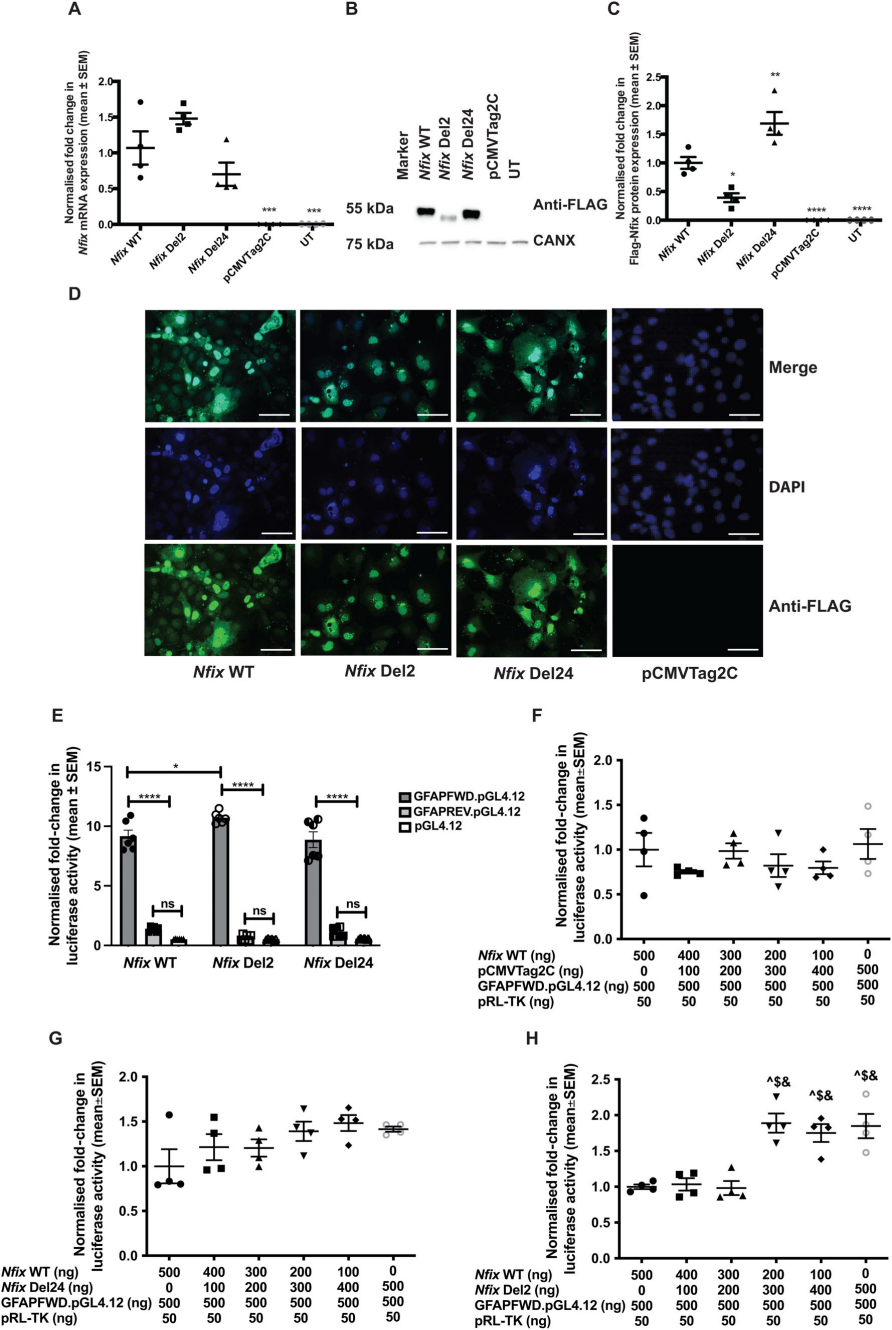
*In vitro* expression assays using wild‐type (WT) and mutant (Del2 and Del24) N‐terminal‐FLAG‐tagged *Nfix* cDNA constructs in COS‐7 cells. COS‐7 cells were transiently transfected with N‐terminal‐FLAG‐tagged WT *Nfix* cDNA constructs (*Nfix* WT), mutant *Nfix* cDNA constructs (*Nfix* Del2 and *Nfix* Del24), or an empty expression vector (pCMVTag2C). Untransfected (UT) COS‐7 cells were used as controls. (*A*) Quantitative real‐time PCR (qRT‐PCR) analysis showed that there was no significant difference in the amount of *Nfix* Del2 or *Nfix* Del24 expression compared to *Nfix* WT. *Gapdh* and *Tbp1* were used as the housekeeping genes against which *Nfix* expression was normalized. (*B*) Western blot analysis using anti‐FLAG antibodies revealed *Nfix* Del2 and *Nfix* Del24 produced smaller NFIX proteins (< 55 kDa) compared to WT (55 kDa). Antibodies against calnexin (CANX) (75 kDa) were used as loading control. (*C*) Relative N‐terminal‐FLAG‐tagged NFIX expression, normalized to CANX expression, was quantified by densitometry analysis. (*D*) Cellular localization of transiently transfected WT and mutant NFIX following immunofluorescence analysis using an anti‐FLAG antibody showed that the nuclear localization of NFIX Del2 and NFIX Del24 was comparable to NFIX WT. (*E*) *In vitro* dual luciferase reporter assays, in which the luciferase reporter gene is under the control of the glial fibrillary acidic protein (*GFAP*) promoter containing three NFIX binding sites, cotransfected with WT or mutant *Nfix* cDNA constructs. The luciferase construct and varying concentrations of *Nfix* WT and (*F*) empty pCMVTag2C vector or (*G*) *Nfix* Del 24 or (*H*) *Nfix* Del2 cDNA constructs were used to cotransfect COS‐7 cells. The *Nfix* WT cDNA construct and the *Nfix* Del 24 mutant construct did not affect the transactivation activity of the NFIX protein at the *GFAP* locus, while the *Nfix* Del 2 mutant construct increased NFIX transactivation activity at the *GFAP* locus in a threshold‐dependent manner. Scale bar = 20 μm. Data are represented as mean ± SEM, *n* = 4–6, *p < 0.05, **p < 0.01, ***p < 0.001, ****p < 0.0001, ns = not significant, ^^^ *p* < 0.01 compared to 500 ng *Nfix* WT, ^$^ *p* < 0.01 compared to 400 ng *Nfix* WT, ^&^ *p* < 0.01 compared to 300 ng *Nfix* WT.

To further investigate the effects of the *Nfix* Del2 and *Nfix* Del24 mutations on *Nfix* transcription, translation, and cellular localization, *in vitro* expression assays in COS‐7 cells transiently transfected with N‐terminal‐FLAG‐tagged WT or mutant (Del2 or Del24) *Nfix* cDNA constructs that retain exon 7 were undertaken. Analysis by qRT‐PCR showed that there was no significant difference in the amount of *Nfix* Del2 or *Nfix* Del24 expression compared to *Nfix* WT, suggesting that these mutations affecting the C‐terminal part of the *Nfix* transcripts were not cleared by NMD mechanisms (Fig. 1*A*). Furthermore, Western blot analysis demonstrated the production of *Nfix* Del2 and *Nfix* Del24 smaller NFIX mutant proteins (<55 kDa), as expected, compared to WT (55 kDa) (Fig. 1*B*), thereby confirming that mutations in the C‐terminal part of the *Nfix* gene produced truncated NFIX proteins. In addition, the expression of the NFIX Del2 protein was significantly decreased (*p* < 0.05), whereas that of the NFIX Del24 protein was significantly increased (*p* < 0.01) compared to NFIX WT, thereby revealing differences in the stabilities and likely degradations of the mutant proteins (Fig. 1*C*). Immunofluorescence analysis showed that the cellular localization of NFIX Del2 and NFIX Del24 proteins was similar to the predominantly nuclear localization of NFIX WT (Fig. 1*D*). The *Nfix* Del140 mutation, which causes skipping of exon 7 to produce the *Nfix* WT ΔEx7 isoform, was not investigated *in vitro*.

To further assess the effects of the *Nfix* Del2 and *Nfix* Del24 mutations on NFIX transcription factor function, given that NFIX is reported to activate *GFAP* expression,^(^ ^24^ ^)^ reporter constructs comprising the luciferase reporter gene downstream of the *GFAP* promoter were transiently cotransfected with WT or mutant *Nfix* cDNA constructs into COS‐7 cells. WT NFIX activated the *GFAP* promoter and caused an approximately ninefold increase (*n* = 4, *p* < 0.0001, Fig. 1*E*) in luciferase activity in cells with the *GFAP* promoter cloned in the forward orientation compared to cells with the *GFAP* promoter cloned in the reverse orientation. Luciferase reporter activity was unaffected by the *Nfix* Del24 mutation (Fig. 1*E*,*G*) compared to WT NFIX (Fig. 1*E*,*F*). In contrast, the *Nfix* Del2 mutation caused a significant increase (*n* = 6, *p* < 0.05, Fig. 1*E*) in luciferase activity, in a threshold‐dependent manner (Fig. 1*H*) compared to WT NFIX, suggesting that the *Nfix* Del2 mutation had aberrant NFIX transactivation activity at the *GFAP* locus. Overall, these findings suggest that different frameshift mutations or in‐frame deletions affecting the C‐terminal part of the *Nfix* gene have distinct consequences on the activity of the resulting mutant NFIX proteins. Therefore, the phenotypes of the three mouse models harboring the *Nfix* allelic variants‐ Del2, Del24, and Del140—were further characterized for features of MSS.

### Phenotypic characterization of *Nfix* Del2, Del24, and Del140 mice

Heterozygous and homozygous *Nfix* mutant and WT littermates were characterized for features of MSS that included abnormalities of growth, skeleton, central nervous system (CNS), viscera, and plasma biochemistry.

#### Analysis of growth, length, and body mass

*Nfix* ^*Del2/Del2*^ mice were characterized by growth retardation and short stature when compared to *Nfix* ^*+/+*^, *Nfix* ^*+/Del2*^, *Nfix* ^*+/Del24*^, *Nfix* ^*Del24/Del24*^, *Nfix* ^*+/Del140*^, and *Nfix* ^*Del140/Del140*^ mice. Thus, at P1, there was no significant difference in the weights of *Nfix* ^*+/+*^(1.5 ± 0.1 g), *Nfix* ^*+/Del2*^ (1.6 ± 0.0 g), and *Nfix* ^*Del2/Del2*^ (1.5 ± 0.1 g) mice (Fig. S4), irrespective of sex, but by P14 the growth rate, measured as weight gained over time, of the *Nfix* ^*Del2/Del2*^ mice was significantly reduced compared to the *Nfix* ^*+/+*^ and *Nfix* ^*+/Del2*^ mice (0.8‐fold, *p* < 0.05, Fig. S5 *A*). In addition, the *Nfix* ^*Del2/Del2*^ mice were visibly smaller than *Nfix* ^*+/+*^ and *Nfix* ^*+/Del2*^ mice at 2 weeks (Fig. S5 *A*). In contrast, the growth rates of the *Nfix* ^*+/Del24*^ and *Nfix* ^*Del24/Del24*^ mice, and the *Nfix* ^*+/Del140*^ and *Nfix* ^*Del140/Del140*^ mice were not significantly different from WT mice between 2 to 12 weeks of age, irrespective of sex (Fig. S5 *B*,*C*). Furthermore, visually, the *Nfix* ^*+/Del24*^, *Nfix* ^*Del24/Del24*^, *Nfix* ^*+/Del140*^, and *Nfix* ^*Del140/Del140*^ mice were indistinguishable from the *Nfix* ^*+/+*^ mice at 12 weeks (Fig. S5 *B*,*C*).

**Fig. 2:**
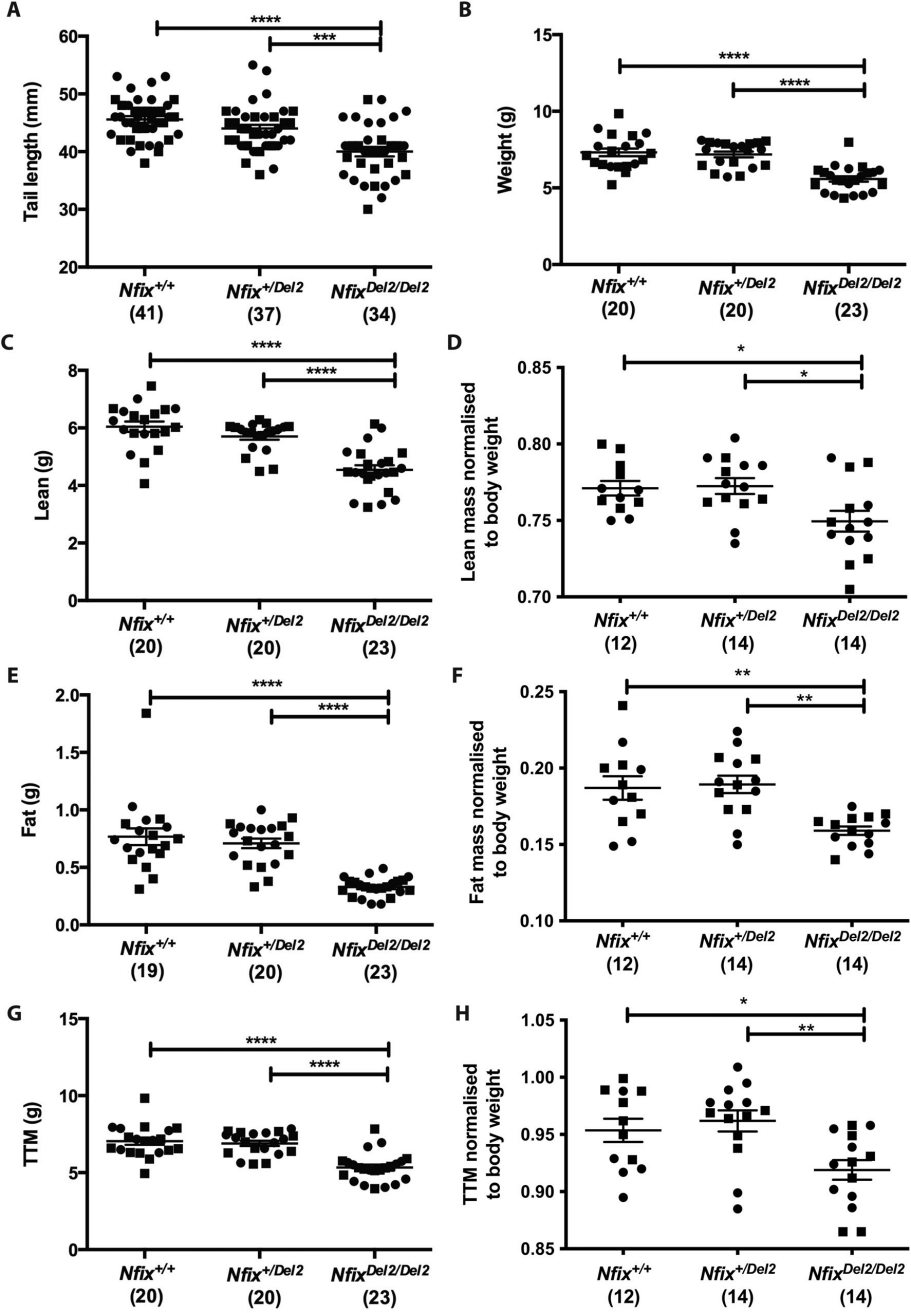
Length and body mass analyses (by echo‐MRI and dual‐energy X‐ray absorptiometry (DXA scan) of *Nfix* ^*+/+*^, *Nfix* ^*+/Del2*^, and *Nfix* ^*Del2/Del2*^ mice at 2–3 weeks of age. (*A*) Tail length, (*B*) total weight, (C) lean mass, (*D*) lean mass normalized to body weight, (*E*) fat mass, (*F*) fat mass normalized to body weight, (*G*) TTM and (*H*) TTM normalized to body weight was significantly reduced in *Nfix* ^*Del2/Del2*^ mice compared to *Nfix* ^*+/+*^ and *Nfix* ^*+/Del2*^ mice. The number of mice analyzed in each case is indicated in parentheses. Data are represented as mean ± SEM, *p < 0.05, **p < 0.01, ***p < 0.001, ****p < 0.0001, circles represent females, squares represent males.

The tail lengths, indicative of vertebral growth, of the *Nfix* ^*Del2/Del2*^ mice were also significantly shorter than *Nfix* ^*+/+*^ and *Nfix* ^*+/Del2*^ mice (0.9‐fold, *p* < 0.001; Fig. 2*A*, Table S2 *A*). In addition, Echo‐MRI analysis revealed a significant decrease in weight (0.8‐fold, *p* < 0.0001, Fig. 2*B*), lean (0.8‐fold, *p* < 0.0001, Fig. 2*C*) and fat mass content (0.4‐fold, *p* < 0.0001, Fig. 2*E*), and dual‐energy X‐ray absorptiometry (DXA) scan analysis revealed a significant decrease in total tissue mass (TTM, (i.e. sum of total lean and total fat mass), 0.8‐fold, *p* < 0.0001, Fig. 2*G*) of *Nfix* ^*Del2/Del2*^ mice compared to *Nfix* ^*+/+*^ and *Nfix* ^*+/Del2*^ mice at 2–3 weeks, in both males and females (Table S2 *A*), even when normalized to body weight (Fig. 2*D*,*F*,*H*). In contrast, the tail lengths, weight, TTM, lean, and fat mass content were not significantly different between *Nfix* ^*+/Del24*^ and *Nfix* ^*Del24/Del24*^ mice, or *Nfix* ^*+/Del140*^ and *Nfix* ^*Del140/Del140*^ mice and WT mice at 12 weeks (Table S2 *B*,*C*).

#### Skeletal and bone turnover analyses

**Fig. 3:**
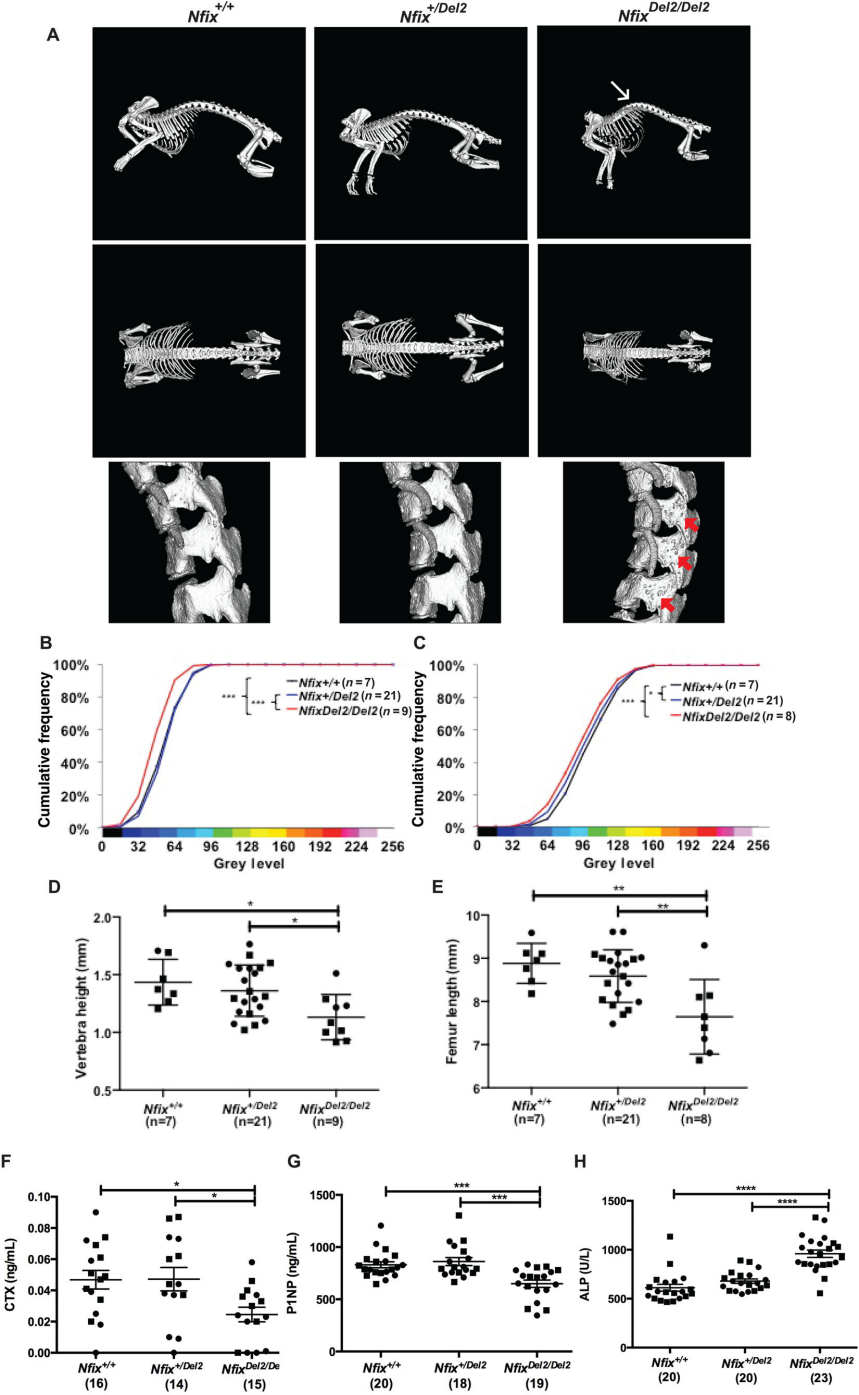
Skeletal abnormalities in *Nfix* ^Del2/Del2^ mice at 2–3 weeks of age. (*A*) Representative MCT scans of skeletons (upper panels) and lumbar and thoracic vertebrae (lower panels) of female *Nfix* ^*+/+*^, *Nfix* ^*+/Del2*^, and *Nfix* ^*Del2/Del2*^ mice. *Nfix* ^*Del2/Del2*^ mice had kyphosis of the thoracolumbar spine (white arrow) and marked porosity (red arrows), which was not present in *Nfix* ^*+/+*^ and *Nfix* ^*+/Del2*^ mice. (*B*–*E*) Faxitron digital X‐ray microradiographic analysis showing cumulative frequency histogram of bone mineral content (BMC) in (*B*) vertebrae and (*C*) femora, and (*D*) vertebral height and (*E*) femoral length from *Nfix* ^*+/+*^, *Nfix* ^*+/Del2*^, and *Nfix* ^*Del2/Del2*^ mice. *Nfix* ^*Del2/Del2*^ mice had reductions in vertebral and femoral BMC and in length. Grayscale images were pseudocolored according to a 16‐color palette in which low mineral content is black and high mineral content is white (Fig. S8). Plasma biochemistry analysis of bone turnover markers in plasma of *Nfix* Del2 mice at 2–3 weeks of age revealed that *Nfix* ^*Del2/Del2*^ mice had (*F*) decreased C‐terminal telopeptide (CTX) concentration, (*G*) reduced procollagen‐type‐1‐N‐terminal propeptide (P1NP) concentration, and (H) raised total alkaline phosphatase (ALP) activity compared to *Nfix* ^*+/+*^ and *Nfix* ^*+/Del2*^ mice. The number of mice analyzed is indicated in parentheses in each case. Data are represented as mean ± SEM, *p < 0.05, **p < 0.01, ***p < 0.001, ****p < 0.0001, circles represent females, squares represent males.

Micro–computed tomography (MCT) (Fig. 3*A*), Alcian Blue, and Alizarin Red staining (Fig. S6) and radiological analyses (Fig. S7 *A*) of skeletons of 2–3 weeks old mice revealed that >30% of *Nfix* ^*Del2/Del2*^ mice had kyphosis compared to <10% of *Nfix* ^*+/+*^ and *Nfix* ^*+/Del2*^ mice. Craniofacial measurements of the skulls of *Nfix* ^*+/+*^, *Nfix* ^*+/Del2*^, and *Nfix* ^*Del2/Del2*^ mice revealed that there was a significant reduction in the skull length of *Nfix* ^*Del2/Del2*^ mice compared to *Nfix* ^*+/+*^ and *Nfix* ^*+/Del2*^ mice (*p* < 0.05; Fig. S8 *A*,*B*). However, there were no significant differences in skull width, nasal bone length, and frontal bone length of *Nfix* ^*Del2/Del2*^ mice, compared to *Nfix* ^*+/+*^ and *Nfix* ^*+/Del2*^ mice (Fig. S8 *C*–*E*), although parietal bone length of *Nfix* ^*Del2/Del2*^ mice was significantly different from that of *Nfix* ^*+/Del2*^ but not *Nfix* ^*+/+*^ mice (*p* < 0.001; Fig. S8 *F*). In contrast, radiological analyses of *Nfix* ^*+/+*^, *Nfix* ^*+/Del24*^, and *Nfix* ^*Del24/Del24*^ mice or *Nfix* ^*+/+*^, *Nfix* ^*+/Del140*^, and *Nfix* ^*Del140/Del140*^ mice revealed no skeletal abnormalities at 12 weeks (Fig. S7 *B*,*C*). MCT analysis of the lumbar and thoracic vertebrae also revealed *Nfix* ^*Del2/Del2*^ mice to have marked porosity at 2–3 weeks (Fig. 3*A*), and faxitron digital X‐ray microradiographic analysis of the caudal vertebrae and femora confirmed significantly reduced BMC of vertebrae and femora (*p* < 0.001; Figs 3*B*,*C*; S9 *A*,*B*) and revealed decreases in vertebral height (0.8‐fold; *p* < 0.05; Fig. 3*D*) and femoral length (0.9‐fold; *p* < 0.01; Fig. 3*E*) in *Nfix* ^*Del2/Del2*^ mice compared to *Nfix* ^*+/+*^ and *Nfix* ^*+/Del2*^ mice at 2 weeks. To explore further the basis of these reductions in vertebral and femoral BMC, osteoclast numbers were assessed by histological analysis of the tibia using tartrate‐resistant acid phosphatase (TRAP) staining (Fig. S9*C*), which did not reveal significant differences in osteoclast numbers between *Nfix* ^*Del2/Del2*^ mice compared to *Nfix* ^*+/+*^ and *Nfix* ^*+/Del2*^ mice at P21 (Fig. S9 *D*,*E*), although the lack of significant difference in the number of osteoclasts could be due to the low number of animals analyzed. To investigate whether the low BMC in *Nfix* ^*Del2/Del2*^ mice may be a result of abnormal osteoclast activity instead, the plasma concentrations of C‐terminal telopeptide (CTX), procollagen‐type‐1‐N‐terminal propeptide (P1NP), and total alkaline phosphatase (ALP) activity, which are markers of bone resorption, bone formation, and bone mineralization, respectively, were therefore measured. *Nfix* ^*Del2/Del2*^ mice, when compared to *Nfix* ^*+/+*^ and *Nfix* ^*+/Del2*^ mice at 2–3 weeks, had reduced CTX concentrations (0.5‐fold, *p* < 0.05, Fig. 3*F*), due to abnormal osteoclast activity, decreased P1NP concentrations (0.8‐fold, *p* < 0.001, Fig. 3*G*), indicating a reduction in bone formation, and raised plasma ALP activity, possibly implying abnormal bone mineralization (1.5‐fold, *p* < 0.0001, Fig. S3 *H*), thereby suggesting an overall abnormal bone turnover phenotype.

#### CNS abnormalities

**Fig. 4:**
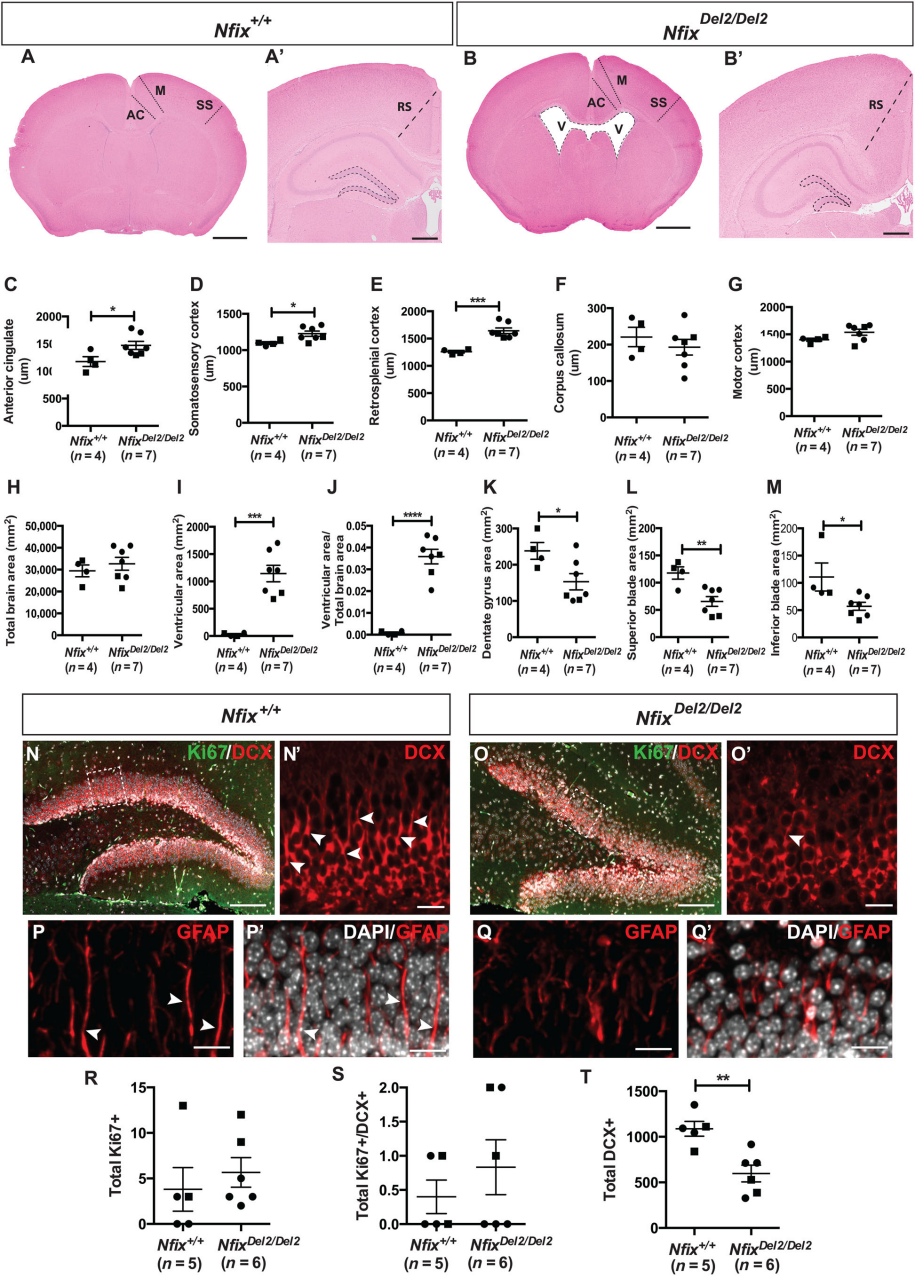
Analyses of neurological abnormalities in *Nfix* Del2 mice at 2–3 weeks of age. Hematoxylin stained coronal brain sections of (*A*) *Nfix* ^*+/+*^ and (*B*) *Nfix* ^*Del2/Del2*^ mice shown at low magnification. Width measurements (black dashed lines shown in (*A*) and (*B*); scale bar = 1300 μm) were taken of the (*C*) cingulate cortex, (*D*) somatosensory cortex, (*F*) corpus callosum, and (*G*) motor cortex using rostral sections. Width measurements (black dashed lines shown in (*A*′) and (*B*′); scale bar = 600 μm) were taken of (*E*) retrosplenial cortex using caudal sections. There was no difference in total brain area (*H*) in *Nfix* ^*Del2/Del2*^ mice compared to *Nfix* ^*+/+*^. (*I*) Ventricular area and (*J*) ventricular area as a proportion of total brain area were significantly larger in *Nfix* ^*Del2/Del2*^ animals compared to *Nfix* ^*+/+*^. Total dentate gyrus area (*K*) was significantly decreased in *Nfix* ^*Del2/Del2*^ animals compared to *Nfix* ^*+/+*^. Both the superior blade area (*L*) and inferior blade (*M*) area (dashed regions in (*A*') and (*B*′)) were significantly decreased in *Nfix* ^*Del2/Del2*^ animals compared to *Nfix* ^*+/+*^. V, ventricle; AC, anterior cingulate cortex; M, motor cortex; RS, retrosplenial cortex; SS, somatosensory cortex. Immunofluorescence labeling of (*N*) *Nfix* ^*+/+*^ and (*O*) *Nfix* ^*Del2/Del2*^ coronal sections with Ki67 (green) and DCX (red). The boxed regions in (*N*) and (*O*) are shown at higher magnification in (*N*′) and (*O*′), respectively. DCX+ processes extended vertically in the *Nfix* ^*+/+*^ (arrowheads in (*N*′)) mice, while DCX+ vertical processes were abnormal in the *Nfix* ^*Del2/Del2*^ animals (arrowhead in (*O*′)). Scale bar = 100 μm in (*N*) and (*O*), 15 μm in (*N*′) and (*O*′). Immunofluorescence labeling of (*P*, *P*′) *Nfix* ^*+/+*^ and (*Q*, *Q*') *Nfix* ^*Del2/Del2*^ coronal sections with glial fibrillary acidic protein (GFAP) (red) and 4′, 6‐diamidino‐2‐phenylindole (DAPI) (white). Radially oriented GFAP+ fibers (arrowheads in (*P*) and(*P*′)) were present in the *Nfix* ^*+/+*^ (*Q*, *Q*') mice. GFAP+ radial processes were malformed in *Nfix* ^*Del2/Del2*^ animals. Scale bar = 15 μm in (*P*), (*P*′), (*Q*), and (*Q*'). There was no change in (*R*) the total number of proliferating cells (Ki67+) and (*S*) the total number of proliferating neuroblasts (Ki67+ DCX+) in *Nfix* ^*Del2/Del2*^ mice compared to *Nfix* ^*+/+*^. (*T*) There were significantly fewer DCX‐labeled neuroblasts within the dentate gyrus of *Nfix* ^*Del2/Del2*^ mice compared to *Nfix* ^*+/+*^. The number of mice analyzed is indicated in parentheses in each case. Data are represented as mean ± SEM, *p < 0.05, **p < 0.01, ***p < 0.001, ****p < 0.0001, circles represent females, squares represent males.

Histological analysis of brains from 23 week‐old *Nfix* ^*+/+*^ (Fig. 4*A*) and *Nfix* ^*Del2/Del2*^ (Fig. 4*B*) mice revealed that *Nfix* ^*Del2/Del2*^ mice had enlarged anterior cingulate (*p* < 0.05; Fig. 4*C*), somatosensory (*p* < 0.05; Fig. 4*D*) and retrosplenial (*p* < 0.01; Fig. 4*E*) cortices. Moreover, both the total area of the ventricular zone (at the level of the corpus callosum) and the ventricular area normalized to the total brain section area were significantly larger in *Nfix* ^*Del2/Del2*^ mice (*p* < 0.001; Fig. 4*J,J*). In contrast, the hippocampal dentate gyrus was significantly smaller (*p* < 0.05; Figs 4*K* and S10*A*) compared to *Nfix* ^*+/+*^ mice. Both the superior blade (*p* < 0.01) and inferior blade areas (*p* < 0.05) of the dentate gyrus (Fig. 4*M,M*) were significantly decreased in *Nfix* ^*Del2/Del2*^ mice compared to *Nfix* ^*+/+*^mice. There was no difference in the size of the corpus callosum, motor cortex, and total brain area in *Nfix* ^*Del2/Del2*^ mice compared to *Nfix* ^*+/+*^mice (Fig. 4*H,H*).

Mice homozygous for a null exon 2 *Nfix* allele were previously reported to exhibit similar phenotypes, including expanded ventricles and smaller dentate gyrus.^(^ ^18^ ^)^ These mice have also been shown to have reduced production of new neurons within the postnatal dentate gyrus.^(^ ^20^ ^)^ To determine whether the *Nfix* ^*Del2/Del2*^ mice exhibited a similar phenotype, we performed co‐immunofluorescence analysis (Fig. 4*N*,*O*) of neuroblasts with the neuroblast marker (doublecortin (DCX)) and proliferating cells (with the marker for proliferation Ki67), as well as with the neural stem cell marker (glial fibrillary acidic protein (GFAP); Fig. 4*P*,*Q*). This analysis revealed no significant difference in either total proliferating cells (Ki67+; Fig. 4*R*) or proliferating neuroblasts (Ki67+; DCX+; Fig. 4*S*) in *Nfix* ^*Del2/Del2*^ mice compared to *Nfix* ^*+/+*^ mice. However, there were significantly fewer neuroblasts in the dentate gyrus of *Nfix* ^*Del2/Del2*^ mice compared to *Nfix* ^*+/+*^ (*p* < 0.01; Fig. 4*T*), indicative of a smaller number of differentiating cells and neurons, similar to reports of a global deficit in neuroblasts observed in homozygous *Nfix* null mice deleted for exon 2.^(^ ^20^ ^)^ The neuroblast vertical processes extended vertically in the granule cell layer in the *Nfix* ^*+/+*^ mice (Fig. 4*N*,*N*') but were abnormal in the neuroblasts of *Nfix* ^*Del2/Del2*^ mice (Fig. 4*O*,*O*'). Similarly, radially oriented GFAP‐labeled fibers corresponding to postnatal neural stem cells (Fig. 4*P*,*P*') were present in the *Nfix* ^*+/+*^ mice, but malformed in the *Nfix* ^*Del2/Del2*^ mice (Fig. 4*Q*,*Q*'). In contrast, histological analysis of the brain of *Nfix* ^*+/+*^, *Nfix* ^*+/Del24*^, and *Nfix* ^*Del24/Del24*^ or *Nfix* ^*+/+*^, *Nfix* ^*+/Del140*^, and *Nfix* ^*Del140/Del140*^ mice at 12 weeks revealed no abnormalities (Fig. S10 *B*,*C*).

#### Extraskeletal abnormalities

Plasma biochemical analysis revealed that *Nfix* ^*Del2/Del2*^ mice, when compared to *Nfix* ^*+/+*^ and *Nfix* ^*+/Del2*^ mice, had raised plasma urea (1.4‐fold, *p* < 0.0001, Fig. S11 *A*) and raised total bilirubin (1.5‐fold, *p* < 0.0001, Fig. S11 *B*) at 2–3 weeks, in males and females (Table S2 *A*), consistent with abnormal kidney and liver function, respectively. However, there were no significant differences in the plasma concentrations of sodium, potassium, chloride, total and corrected calcium, inorganic phosphate, aspartate aminotransferase, alanine aminotransferase, albumin, creatinine, and creatine kinase in the *Nfix* ^*Del2/Del2*^ mice compared to the *Nfix* ^*+/+*^ and *Nfix* ^*+/Del2*^ mice (Table S2 *A*). Moreover, histology of the kidneys and livers from *Nfix* ^*Del2/Del2*^ mice revealed no abnormalities compared to *Nfix* ^*+/+*^ and *Nfix* ^*+/Del2*^ mice (Fig. S10 *A*). In addition, histological examination for liver inflammation (Fig. S11 *C*.*E*.*F*) and liver fibrosis (Fig. S11 *D*,*G*) revealed no hepatic abnormalities in the *Nfix* ^*Del2/Del2*^ mice compared to the *Nfix* ^*+/+*^ and *Nfix* ^*+/Del2*^ mice. The plasma biochemistry of *Nfix* ^*+/+*^, *Nfix* ^*+/Del24*^, *Nfix* ^*Del24/Del24*^, *Nfix* ^*+/Del140*^, and *Nfix* ^*Del140/Del140*^ mice at 12 weeks was similar (Supplemental Table S2 *B*,*C*), suggesting normal kidney and liver function in these mice, and histological analysis of the liver, kidney, lung, and heart of these mice revealed no abnormalities (Fig. S10 *B*,*C*).

### Effects of the three exon 7 mutations on the expression of *Nfix* paralogs in *Nfix* Del2, *Nfix* Del24, and *Nfix* Del140 MEFs

**Fig. 5:**
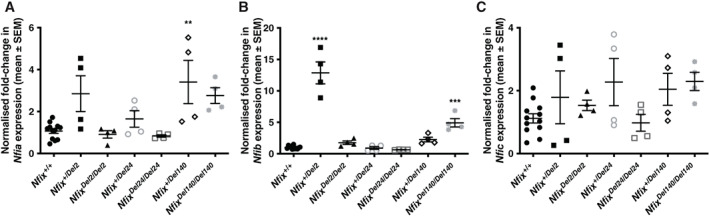
*Nfia*, *Nfib*, and *Nfic* gene expression in *Nfix* Del2, *Nfix* Del24, and *Nfix* Del140 MEFs. qRT‐PCR analysis in *Nfix* ^*+/+*^, *Nfix* ^*+/Del2*^, *Nfix* ^*Del2/Del2*^, *Nfix* ^*+/Del24*^, *Nfix* ^*Del24/Del24*^, *Nfix* ^*+/Del140*^, and *Nfix* ^*Del140/Del140*^ MEFs of (*A*) *Nfia*, (*B*) *Nfib*, and (*C*) *Nfic* expression, with *Gapdh* and *Canx* used as the housekeeping genes against which candidate gene expression was normalized. Data are represented as mean ± SEM, *n* = 4–12, *p < 0.05, **p < 0.01, ***p < 0.001, ****p < 0.0001 compared to mean of *Nfix* ^*+/+*^ MEFs.

Since the variability in the phenotypes in the *Nfix* mouse models could be due to functional redundancy provided by the other members of the *NFI* gene family of transcription factors, we pursued RNA sequencing analysis to investigate differences in *Nfia*, *Nfib*, and *Nfic* ^(^ ^25^, ^26^ ^)^ gene expression in the MEFs derived from the *Nfix* ^*+/+*^, *Nfix* ^*+/Del2*^, *Nfix* ^*Del2/Del2*^, *Nfix* ^*+/Del24*^, *Nfix* ^*Del24/Del24*^, *Nfix* ^*+/Del140*^, and *Nfix* ^*Del140/Del140*^ mice. RNA sequencing analysis identified that, compared to the mean of *Nfix* ^*+/+*^ MEFs, *Nfia* transcripts were significantly altered (≥2‐fold‐change; *p* < 0.05; Table 2) in the *Nfix* ^*+/Del140*^ (2.28‐fold‐change; *p* = 5.37 × 10^−10^; Table 2) and *Nfix* ^*Del140/Del140*^ MEFs (2.19‐fold‐change; *p* = 3.84 × 10^−9^; Table 2), while *Nfib* transcripts were significantly altered in the *Nfix* ^*+/Del2*^ (6.27‐fold‐change; *p* = 1.11 × 10^−70^; Table 2) and *Nfix* ^*Del140/Del140*^ MEFs (3.53‐fold‐change; *p* = 4.30 × 10^−28^; Table 2). However, there was no significant change in *Nfic* expression between WT and mutant MEFs (Table 2). To validate the RNA sequencing results, we performed qRT‐PCR analysis using total RNA isolated from the MEFs of *Nfix* ^*+/+*^, *Nfix* ^*+/Del2*^, *Nfix* ^*Del2/Del2*^, *Nfix* ^*+/Del24*^, *Nfix* ^*Del24/Del24*^, *Nfix* ^*+/Del140*^, and *Nfix* ^*Del140/Del140*^ mice. qRT‐PCR analysis confirmed that, compared to the mean expression in the *Nfix* ^*+/+*^ MEFs, *Nfia* expression was significantly increased only in the *Nfix* ^*+/Del140*^ MEFs (3.18‐fold, *p* < 0.01, Fig. 5*A*), while *Nfib* expression was significantly increased in the *Nfix* ^*+/Del2*^ (12.5‐fold‐change, *p* < 0.0001, Fig. 5*B*) and *Nfix* ^*Del140/Del140*^ MEFs (4.79‐fold‐change; *p* < 0.001, Fig. 5*B*), consistent with the RNA sequencing data. Moreover, in agreement with the RNA sequencing results, there was no significant change in *Nfic* expression in the mutant MEFs compared to WT (Fig. 5*C*). Therefore, our results suggest that in the *Nfix* ^*+/Del2*^, *Nfix* ^*+/Del140*^, and *Nfix* ^*Del140/NfixDel140*^ mice, *Nfia* and *Nfib* but not *Nfic* change their expression pattern in order to potentially compensate for their respective *Nfix* Del2 and Del140 frameshift mutations, which could possibly explain the lack of abnormalities in these mice, while *Nfia*, *Nfib*, and *Nfic* expression was unaltered in the unaffected *Nfix* ^*+/Del24*^ and *Nfix* ^*Del24/Del2*4^ mice, thereby suggesting that the in‐frame *Nfix* Del2 mutations might potentially be tolerated and is probably not as damaging as a frameshift mutation. This is in contrast to *Nfix* ^*Del2/Del2*^ mice where unchanged *Nfia*, *Nfib*, or *Nfic* gene expression suggests a lack of functional redundancy from the *Nfix* paralogs in the homozygous *Nfix* Del2 mice, which might possibly account for the more severe phenotype observed in the *Nfix* ^*Del2/Del2*^ mice.

**Table 2:** Fold‐Change in *Nfia*, *Nfib*, and *Nfic* Expression in *Nfix* ^*+/Del2*^, *Nfix* ^*Del2/Del2*^, *Nfix* ^*+/Del24*^, *Nfix* ^*Del24/Del24*^, *Nfix* ^*+/Del140*^, and *Nfix* ^*Del140/Del140*^ Murine Embryonic Fibroblasts (MEFs) Compared with Mean of *Nfix* ^*+/+*^ MEFs as Determined by RNA Sequencing Analysis

Compared to *Nfix* ^*+/+*^	*Nfia*	*Nfib*	*Nfic*
*Nfix* ^*+/Del2*^	1.97 (2.52 × 10^−7^)^a^	6.27 (1.11 × 10^−70^)	1.11 (0.36)
*Nfix* ^*Del2/Del2*^	0.97 (0.86)	1.25 (0.04)	0.97 (0.79)
*Nfix* ^*+/Del24*^	1.10 (0.52)	0.57 (5.49 × 10^−6^)	0.93 (0.49)
*Nfix* ^*Del24/Del24*^	0.96 (0.80)	0.58 (1.03 × 10^−5^)	0.94 (0.59)
*Nfix* ^*+/Del140*^	2.28 (5.37 × 10^−10^)	1.84 (1.41 × 10^−7^)	0.88 (0.27)
*Nfix* ^*Del140/Del140*^	2.19 (3.84 × 10^−9^)	3.53 (4.30 × 10^−28^)	0.97 (0.81)

^a^ Fold‐change with *p*‐values shown in parentheses.

## Discussion

Our study reports the phenotypic characterization of three CRISPR‐Cas9 generated mouse models with three allelic variants in *Nfix* exon 7, which is the most commonly mutated exon in MSS patients. These three allelic mutations, all of which affect the C‐terminal regions of NFIX, have different effects on the phenotypes and on the expression of *Nfix* transcripts and proteins. Thus, of the *Nfix* Del2, *Nfix* Del24, and *Nfix* Del140 mouse models, only the *Nfix* ^*Del2/Del2*^ mice developed postnatal skeletal and cranial defects, brain abnormalities, and likely dysfunction of the kidney and liver, which might potentially account for their premature deaths by 2–3 weeks of age, whereas the *Nfix* ^*+/Del2*^, *Nfix* ^*+/Del24*^, *Nfix* ^*Del24/Del24*^, *Nfix* ^*+/Del140*^, and *Nfix* ^*Del140/Del140*^ mice were viable, normal, and fertile and survived to adulthood. These observations indicate that allelic variation, rather than potential off‐target effects of the CRISPR‐Cas9 system, could be responsible for the differences in phenotypes in the three *Nfix* mouse models, despite similar allelic mutations, the same environmental conditions, and identical genetic background, which is a common occurrence even on inbred backgrounds.^(^ ^27^, ^28^ ^)^ Only the two‐nucleotide deletion in *Nfix* Del2 mice caused a frameshift and the introduction of a premature stop codon, which led to the production of intermediate levels of mutant NFIX Del2 protein with aberrant NFIX protein function that might potentially account for the more severe phenotype observed in the *Nfix* ^*Del2/Del2*^ mice, while the 24‐nucleotide in‐frame deletion in *Nfix* Del24 mice caused the loss of eight amino acids, which could potentially be tolerated and is probably not as damaging as a frameshift mutation, whereas the 140‐nucleotide deletion in the *Nfix* Del140 mice, comprising 53 nucleotides from exon 7 and 87 nucleotides of intron 7 including the splice donor site, caused skipping of exon 7 and alternative splicing of exon 6 to exon 8 to produce WT (normal) *Nfix* isoforms. This suggests that different frameshift mutations or in‐frame deletions affecting the C‐terminal part of the *Nfix* gene have different consequences on the transcripts and activity of the resulting proteins, thereby accounting for the different phenotypes in the three mouse models.

Cell autonomous, monoallelic, and stochastic variation in gene expression, as well as functionally redundant paralogs, could also account for phenotypic variability.^(^ ^25^, ^26^, ^29^, ^30^, ^31^ ^)^ Redundant paralogs that are ubiquitously expressed in a partially overlapping manner and that recognize similar motifs may provide backup for one another in case of mutation by changing their expression pattern and acquiring new regulatory capabilities in order to compensate for the mutation. For example, NFIA, NFIB, NFIC, and NFIX have the same conserved N‐terminal DNA binding and dimerization domain that enables all four related genes to recognize the same consensus sequence present in the promoter region of genes expressed in almost every organ, including the brain, lung, liver, intestine, and skeleton. *Nfia* ^*−/−*^ mice have CNS and kidney abnormalities^(^ ^32^ ^)^ and die perinatally,^(^ ^33^ ^)^ *Nfib* ^*−/−*^ mice have CNS and lung anomalies and die at birth,^(^ ^34^ ^)^ while *Nfic* ^*−/−*^ mice have only a mild phenotype involving abnormal tooth development of the incisors and molars.^(^ ^35^ ^)^ More recently, overlapping patterns of NFIA, NFIB, and NFIX expression have been reported in the brain.^(^ ^36^ ^)^ NFIA, NFIB, NFIC, and NFIX, which were previously shown to interact with each other as well as other cofactors, bind the same regulatory motif of promoters of genes, such as brain fatty acid binding protein (*B*‐*FABP*), *GFAP*, and inscuteable (*INSC*), and the NFIs or the ratio of the four NFIs have been shown to act either antagonistically or synergistically to regulate transcription in a promoter and context dependent manner.^(^ ^37^, ^38^, ^39^ ^)^ Moreover, knockdown of one NFI member can affect the expression levels of other NFI members, suggesting cross‐talks and possible compensation within the NFI family.^(^ ^37^ ^)^ NFIX was also recently shown to act sequentially after NFIA and NFIB during gliogenesis within the spinal cord, and NFIB was reported to be able to activate *Nfix* expression *in vitro*, thereby suggesting autoregulatory mechanisms within the NFI gene family.^(^ ^38^ ^)^ In this study, we have shown that the combination of NFI family expression might potentially influence the phenotypes of the *Nfix* mouse models. *Nfia* and *Nfib*, but not *Nfic*, change their expression pattern in order to possibly compensate for their respective *Nfix* Del2 and Del140 frameshift mutations in the unaffected *Nfix* ^*+/Del2*^, *Nfix* ^*+/Del140*^, and *Nfix* ^*Del140/NfixDel140*^ mice, while *Nfia*, *Nfib*, and *Nfic* expression was unaltered in the unaffected *Nfix* ^*+/Del24*^ and *Nfix* ^*Del24/Del2*4^ mice, thus suggesting that the in‐frame *Nfix* Del24 mutations might potentially be tolerated and are probably not as damaging as a frameshift mutation. Moreover, the lack of functional redundancy from the *Nfix* paralogs in the *Nfix* ^*Del2/Del2*^ mice as well as the presence of intermediate levels of aberrant mutant NFIX Del2 protein might possibly account for the more severe phenotype observed in the *Nfix* ^*Del2/Del2*^ mice.

*Nfix* ^*Del2/Del2*^ mice represent a mouse model for MSS in which patients commonly have: reduced growth rate; short stature; craniofacial defects; osteopenia with increased fracture rate and kyphosis that normally worsens in puberty and adolescence and that is possibly aggravated by decreased bone density;^(^ ^15^ ^)^ and anxiety and intellectual disability due to nonspecific rain abnormalities.^(^ ^1^, ^2^ ^)^ Thus, the *Nfix* ^*Del2/Del2*^ mice had; short stature; reduced growth and TTM; kyphosis; shortened skull; marked porosity of the vertebrae; reduced BMC; shorter vertebrae height and femur length; reduced plasma CTX and P1NP concentrations but increased total ALP activity, indicative of abnormal bone function; and raised plasma urea and total bilirubin levels, suggestive of renal and hepatic dysfunction, which merits further investigation. Furthermore, *Nfix* ^*Del2/Del2*^ mice had enlarged anterior cingulate, somatosensory and retrosplenial cortices, and ventricles but reduced dentate gyrus (Table S1). However, other features present in MSS patients, which include intellectual disability, airway obstruction leading to respiratory problem, umbilical hernia, cardiac anomalies, and abnormal bone maturation,^(^ ^1^, ^2^, ^3^, ^15^, ^16^, ^40^ ^)^ were not assessed in the *Nfix* ^*Del2/Del2*^ mice in this study. Plasma biochemistry in MSS patients is reported to be usually normal, and our findings of elevated urea and bilirubin concentrations and ALP activity in association with reductions in plasma CTX and P1NP concentrations in the *Nfix* ^*Del2/Del2*^ mice may represent important differences to MSS patients, or it may be that such abnormalities do occur in MSS patients but have hitherto not been found. This latter notion is a possibility as exemplified by our experience. Thus, following our identification of likely renal dysfunction in the *Nfix* ^*Del2/Del2*^ mice, ultrasound scan investigations were undertaken in two MSS patients and revealed the occurrence of renal cysts in both patients and nephrocalcinosis in one (Hennekam–personal communication). Moreover, the reduction in plasma CTX concentrations in the *Nfix* ^*Del2/Del2*^ mice may suggest abnormal osteoclast activity and function, which merits further investigation. Moreover, the paradoxical increased plasma ALP activity, which is a marker of bone turnover, in association with reduced plasma concentrations of CTX and P1NP, which are markers of bone resorption and bone formation respectively, in the *Nfix* ^*Del2/Del2*^ mice suggests additional extraskeletal origin for the raised ALP activity such as the kidneys or intestine, but not liver as mice, in contrast to humans, express little or no ALP in the liver,^(^ ^41^ ^)^ and a search for additional renal or intestinal abnormalities in MSS may be warranted. Thus, it seems possible that MSS patients may have renal, intestinal, and hepatic dysfunction, and that there may be more similarities with the *Nfix* ^*Del2/Del2*^ mice.

Our *Nfix* ^*Del2/Del2*^ mice have similarities and differences when compared to two previous homozygous *Nfix*‐deficient mouse models that had targeted deletions of exon 2^(^ ^17^, ^18^ ^)^ (Table S1). Thus, homozygous *Nfix‐*deficient mice (*Nfix* ^*lacZ/lacZ*^) were viable and had: growth retardation; an inability to fully open eyes; ataxic gait; feet‐clasping posture when lifted by their tail indicating neurological abnormalities; gastrointestinal defects; brain malformations consisting of hydrocephalus and partial agenesis of the corpus callosum; defects in endochondral ossification, reduction in trabecular bone formation and calcification; thinning of cranial bones; kyphotic deformation of the spine; and early postnatal death between 3 and 4 weeks of age.^(^ ^17^ ^)^ The other homozygous *Nfix* ^*−/−*^ mice showed; failure to thrive and grow when on a standard lab chow diet; delayed eye and ear opening; leg‐clasping phenotypes indicating neuroanatomical defects; increased brain weight due to expansion of the cortex and entire brain along the dorsal ventral axis; aberrant neocortex, cerebellum, hippocampus, and spinal cord formation; and an abnormal ventricular cell population due to excessive generation of *Pax6*‐expressing ventricular cells with hydrocephalus.^(^ ^18^, ^20^, ^38^, ^39^, ^42^, ^43^, ^44^, ^45^, ^46^ ^)^ Liver and kidney phenotypes were not assessed in these two previously reported *Nfix*‐deficient mouse models, although it is important to note that *Nfix* ^*lacZ/lacZ*^ mice had gastrointestinal defects.^(^ ^17^ ^)^ Importantly, the *Nfix* ^*Del2/Del2*^ mice are not *Nfix*‐deficient but instead have aberrant *Nfix* transcripts that escape NMD and lead to the production of mutant truncated NFIX protein, which is representative of MSS. Interestingly, MSS patients are heterozygous for *NFIX* mutations, and this contrasts with *Nfix* ^*+/Del2*^ mice, which are normal, while developmental, skeletal, cranial, neural, hepatic, and renal abnormalities are observed in *Nfix* ^*Del2/Del2*^ mice, which could account for their reduced viability. However, phenotypic differences between organisms are not uncommon and can be attributed to allelic variation, modifier genes, genetic variations, genetic background, environmental conditions, and reduced sensitivity of assays, such as behavioral assays, in animal models *versus* in patients.^(^ ^29^, ^30^, ^31^ ^)^ For example, the autosomal dominant disorder spondyloepimetaphyseal dysplasia, Missouri type (SEMD_MO_) in humans, is due to a heterozygous matrix metalloproteinase 13 (*MMP13*) missense F56S mutation, whereas heterozygous *Mmp13* ^*+/−*^ mice deleted for exons 3, 4, and 5 have normal growth plates, but the homozygous *Mmp13* ^*−/−*^ mice have defects in growth plate cartilage and delayed endochondral ossification.^(^ ^47^ ^)^

In summary, in this study we report three *Nfix* mouse models with three different targeted mutations in exon 7 of the *Nfix* gene, which are representative of the most frequent *NFIX* mutations observed in MSS patients. The three mouse models, although being on the same genetic background, have differing phenotypes and viability. While the *Nfix* ^*Del2/Del2*^ mice have some similarities to previously reported *Nfix* deficient mouse models, they also have a number of other phenotypes that are consistent with MSS. Further studies of the *Nfix* ^*Del2/Del2*^ mice will help better understand the role of *NFIX* mutations that result in dominant‐negative NFIX proteins and give rise to MSS, as well as provide useful resources for testing potential future treatments.

## Author Contributions

**Kreepa G. Kooblall:** Conceptualization; data curation; formal analysis; investigation; methodology; validation; writing – original draft; writing – review and editing. **Mark Stevenson:** Supervision; writing – original draft; writing – review and editing. **Michelle Stewart:** Data curation; methodology; project administration; writing – review and editing. **Lachlan Harris:** Conceptualization; data curation; formal analysis; investigation; methodology; validation; writing – review and editing. **Oressia Zalucki:** Conceptualization; data curation; formal analysis; investigation; methodology; validation; writing – review and editing. **Hannah Dewhurst:** Data curation; formal analysis; investigation; methodology; validation; writing – review and editing. **Natalie Butterfield:** Data curation; formal analysis; investigation; methodology; validation; writing – review and editing. **Houfu Leng:** Data curation; formal analysis; investigation; methodology; validation; writing – review and editing. **Tertius A. Hough:** Data curation; investigation; methodology; validation; writing – review and editing. **Da Ma:** Data curation; formal analysis; investigation; methodology; software; validation; writing – review and editing. **Bernard Siow:** Conceptualization; data curation; formal analysis; investigation; methodology; software; validation; writing – review and editing. **Paul Potter:** Writing – review and editing. **Roger D. Cox:** Writing – review and editing. **Stephen D.M. Brown:** Writing – review and editing. **Nicole Horwood:** Supervision; writing – review and editing. **Benjamin Wright:** Data curation; formal analysis; investigation; methodology; software; validation; writing – review and editing. **Helen Lockstone:** Conceptualization; data curation; formal analysis; investigation; methodology; software; validation; writing – review and editing. **David Buck:** Software; supervision; writing – review and editing. **Tonia Vincent:** Supervision; writing – review and editing. **Fadil M. Hannan:** Conceptualization; writing – review and editing. **J.H. Duncan Bassett:** Conceptualization; funding acquisition; supervision; writing – review and editing. **Graham R. Williams:** Conceptualization; funding acquisition; supervision; writing – review and editing. **Kate E. Lines:** Supervision; writing – original draft; writing – review and editing. **Michael Piper:** Conceptualization; funding acquisition; supervision; writing – review and editing. **Sara Wells:** Resources; writing – review and editing. **Lydia Teboul:** Conceptualization; data curation; formal analysis; investigation; methodology; resources; supervision; validation; writing – review and editing. **Raoul C. Hennekam:** Conceptualization; funding acquisition; supervision; writing – review and editing. **Rajesh V. Thakker:** Conceptualization; funding acquisition; resources; supervision; writing – original draft; writing – review and editing.

### Peer Review

The peer review history for this article is available at https://www.webofscience.com/api/gateway/wos/peer-review/10.1002/jbm4.10739.

## Supporting information


**Data S1.** Supporting Information.

**Data S2.** Supporting Information.
