# Supplementary material for: A Mouse Model with a Frameshift Mutation in the Nuclear Factor I/X ( NFIX ) Gene Has Phenotypic Features of Marshall‐Smith Syndrome
Source: JBMR Plus. 2023 Mar 30;7(6):e10739. doi: 10.1002/jbm4.10739 (PMC10241085; doi:10.1002/jbm4.10739)
Supplement: Supplementary file 1 — Data S1. Supporting Information. [file JBM4-7-e10739-s001.pdf]

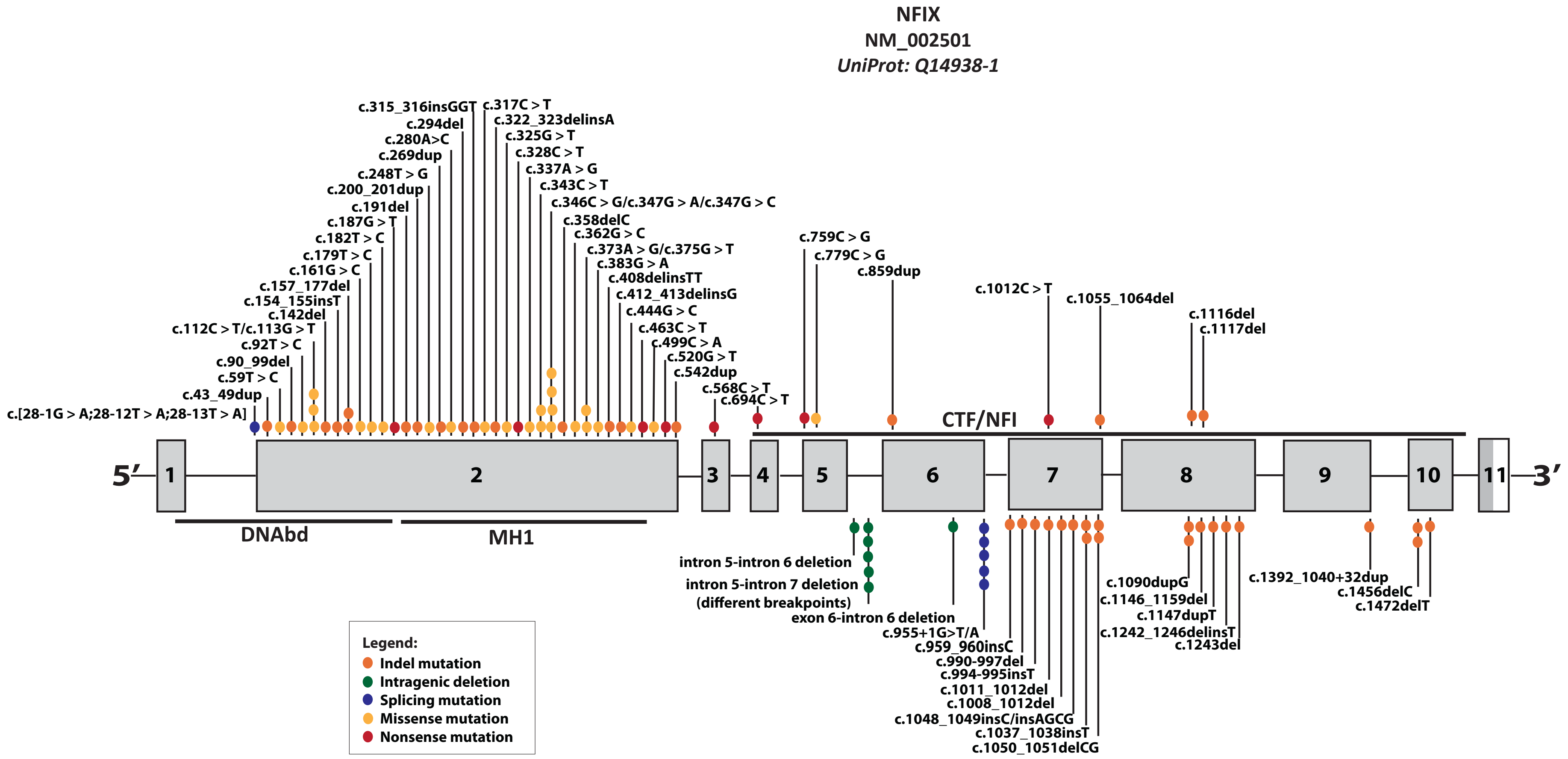

Supplemental Figure S1. Schematic representation of *NFIX* mutations reported in MSS and MAL patients. Structure of wild-type *NFIX* gene, consisting of 11 exons (boxes) comprising a N-terminal DNA binding (DNAbd) and dimerization (MH1) domain and a C-terminal transactivation or repression (CTF/NFI) domain. In MSS patients, the mutations are clustered in exons 6 to 10 of the *NFIX* gene (vertical bars underneath the gene illustrate positions of mutations) and introduce frame shifts and splice site variants. In MAL patients, the missense, nonsense

and frameshift mutations are predominantly located in exon 2 of the *NFIX* gene (vertical bars above the gene illustrate positions of mutations). The displayed variants refer to recently published original and review papers[1,2].

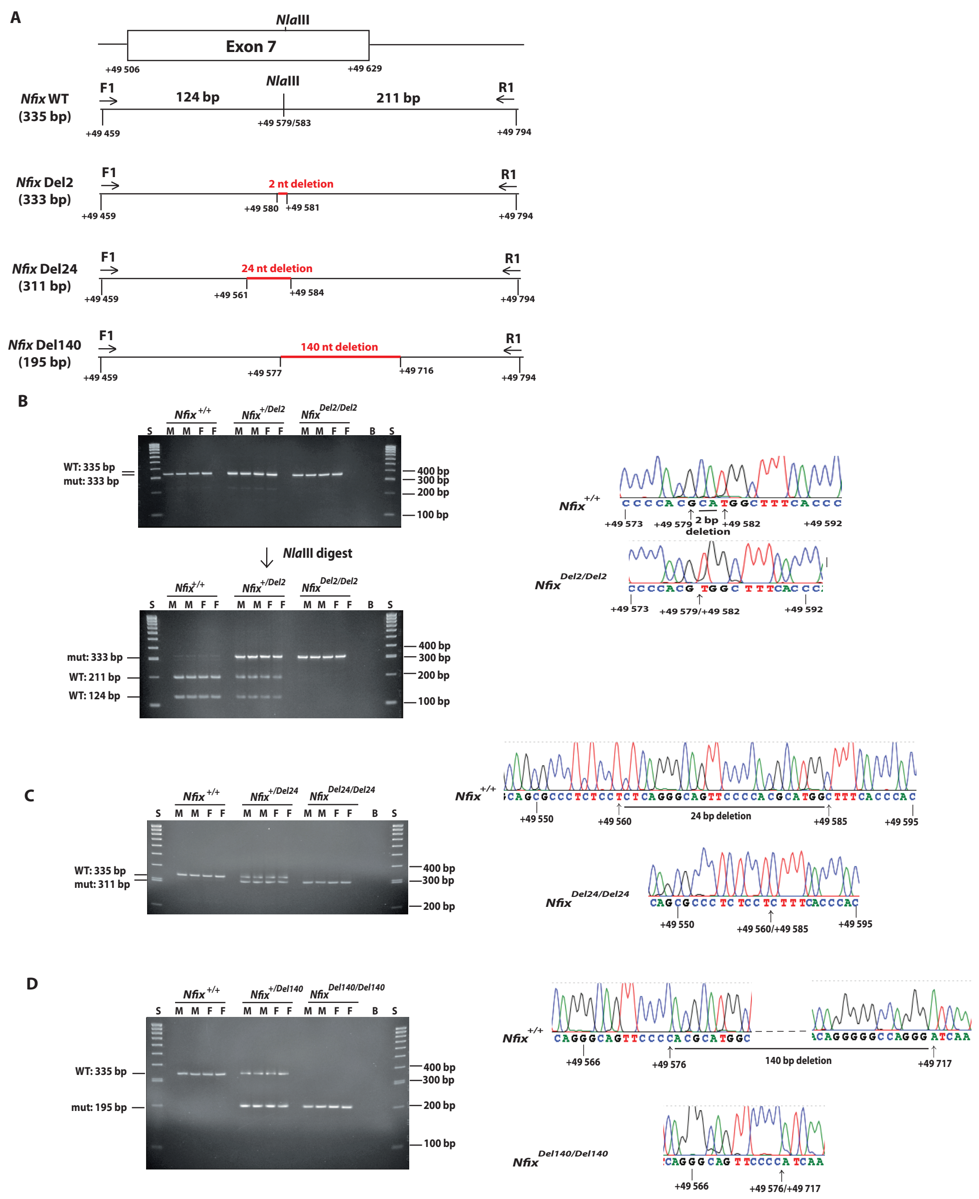

Supplemental Figure S2. Genotyping analysis of *Nfix* mutations generated using the CRISPR/Cas9 system in the three mouse lines. (A) Schematic representation of *Nfix* exon 7 showing: the locations of the PCR primers (F1 and R1) used in the genotyping analysis; and the locations of the deleted nucleotides in the three mouse lines, which coincides with an *Nla*III restriction endonuclease site in each case. Genotyping was performed using genomic DNA samples from *Nfix* Del2, *Nfix* Del24 and *Nfix* Del140 mice. (B) In *Nfix*$^{+/+}$ mice, a 335 bp PCR product (WT) was produced and the sequence corresponded with the consensus exon 7 of murine *Nfix* sequence (ENSMUSG00000001911.16). *Nla*III restriction endonuclease digest of

the 335bp PCR product from *Nfix*$^{+/+}$mice resulted in the formation of 124 bp and 211 bp products. In *Nfix*$^{Del2/Del2}$ mice, a shorter mutant PCR band (Mut) of 333 bp was produced, which was not resolvable from the WT 335 bp band, and which remained undigested by *Nla*III. DNA Sanger sequencing confirmed the location of the 2nt deletion at position +49,580-49,581. In *Nfix*$^{+/Del2}$ mice *Nla*III digestion gave rise to both WT and Mut products. (C) In *Nfix*$^{Del24/Del24}$ mice a shorter Mut band of 311 bp was produced, which could be resolved from the WT 335 bp band. DNA Sanger sequencing confirmed the location of the 24nt deletion at position +49,561-49,584. (D) In *Nfix*$^{Del140/Del140}$ mice a shorter Mut band of 195 bp was produced. DNA Sanger sequencing confirmed the location of the 140nt deletion at position +49,577-49,716. S: 100 bp size marker; B: blank; M: male; F: female.

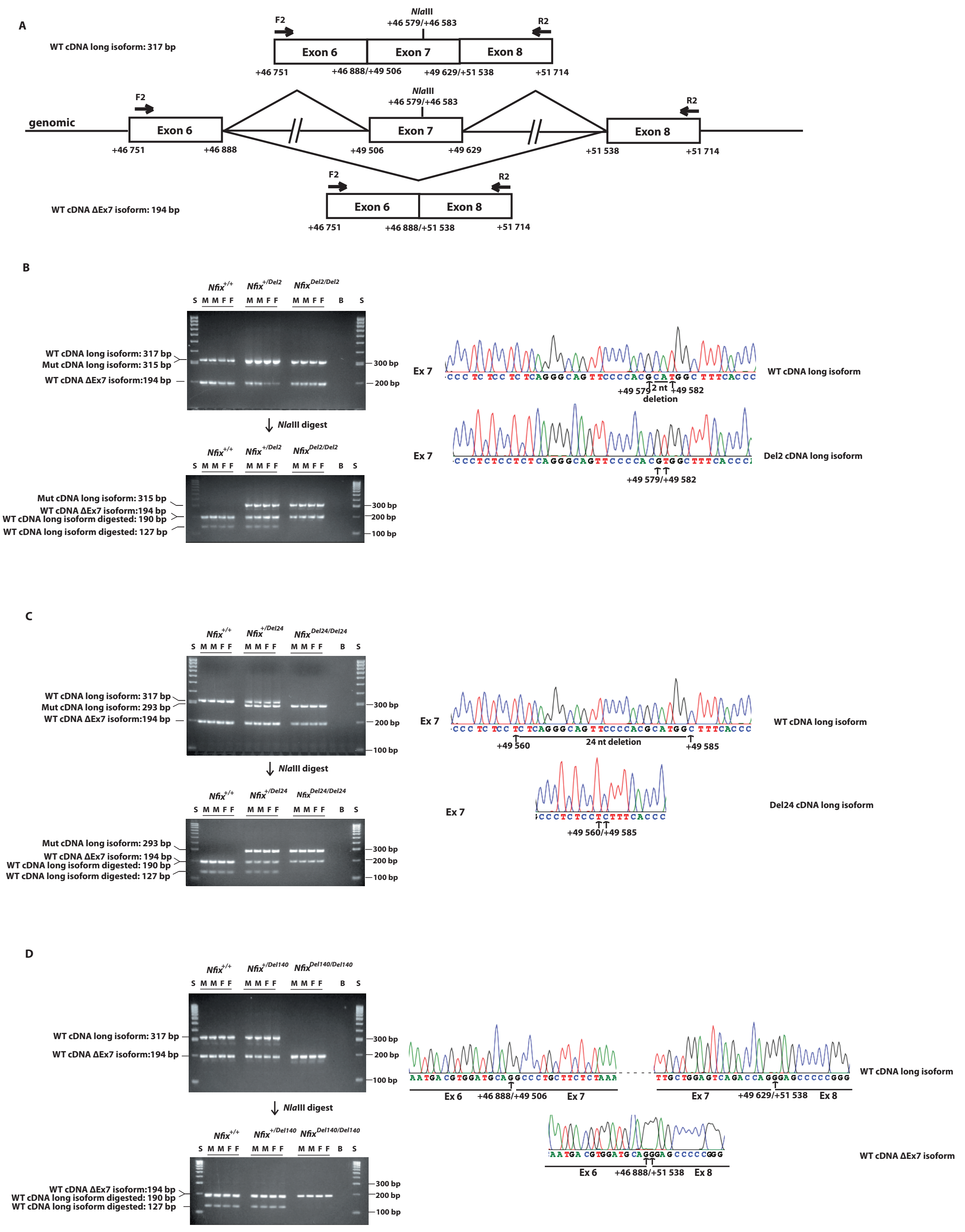

Supplemental Figure S3. Analysis of the effects of *Nfix* mutations on the splicing of exon 7. (A) Schematic representation of alternative splicing of exon 7 from the genomic *Nfix* sequence showing the production of either a wild-type long transcript (WT cDNA long isoform) that retains exon 7 (shown above the genomic locus) or a wild-type short transcript

(WT cDNA ΔEx7 isoform) that lacks exon 7 (shown below the genomic locus). Primers (F2 and R2) homologous to regions located in exons 6 and 8 allowed the PCR amplification of cDNA fragments that comprised both WT cDNA long and WT cDNA ΔEx7 isoforms. (B) In *Nfix*$^{+/+}$ mice, a 317 bp WT cDNA long isoform PCR product and 194 bp WT cDNA ΔEx7 isoform product were produced. In *Nfix*$^{Del2/Del2}$ mice, a 315 bp mutant (Mut) cDNA long isoform product and 194 bp WT cDNA ΔEx7 isoform product were produced. Following *Nla*III digestion the 317 bp WT cDNA long isoform gave rise to 190 bp and 127 bp products whilst the 315 bp mutant cDNA long isoform remained undigested. Sanger sequencing of the WT and Del2 cDNA long isoforms isolated from the gel confirmed the absence of the 2nt at position +49,580-49,581 from this transcript. (C) In *Nfix*$^{Del24/Del24}$ mice, a 293 bp Mut cDNA long isoform PCR product and 194 bp WT cDNA ΔEx7 isoform product were produced. Following *Nla*III digestion the 293bp mutant cDNA long isoform remained undigested. Sanger sequencing of the Del24 cDNA long isoform confirmed the absence of the 24nt at position +49,561-49,584 in this transcript. (D) In *Nfix*$^{Del140/Del140}$ mice, only the 194 bp WT cDNA ΔEx7 isoform PCR product was produced, which was unaffected by *Nla*III digestion. Sanger sequencing of the WT cDNA ΔEx7 isoform confirmed the absence of nucleotides from exon 7 in this transcript. S: 100 bp marker; B: blank.

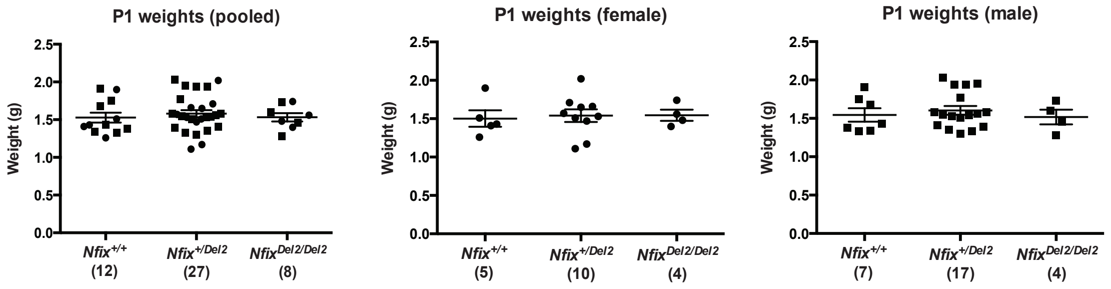

Supplemental Figure S4. Weights of *Nfix* Del2 mice at P1. The weights of $Nfix^{+/+}$, $Nfix^{+/Del2}$ and $Nfix^{Del2/Del2}$ mice. The number of mice analyzed is indicated in parentheses in each case. Data are represented as mean±SEM, circles represent females, squares represent males.

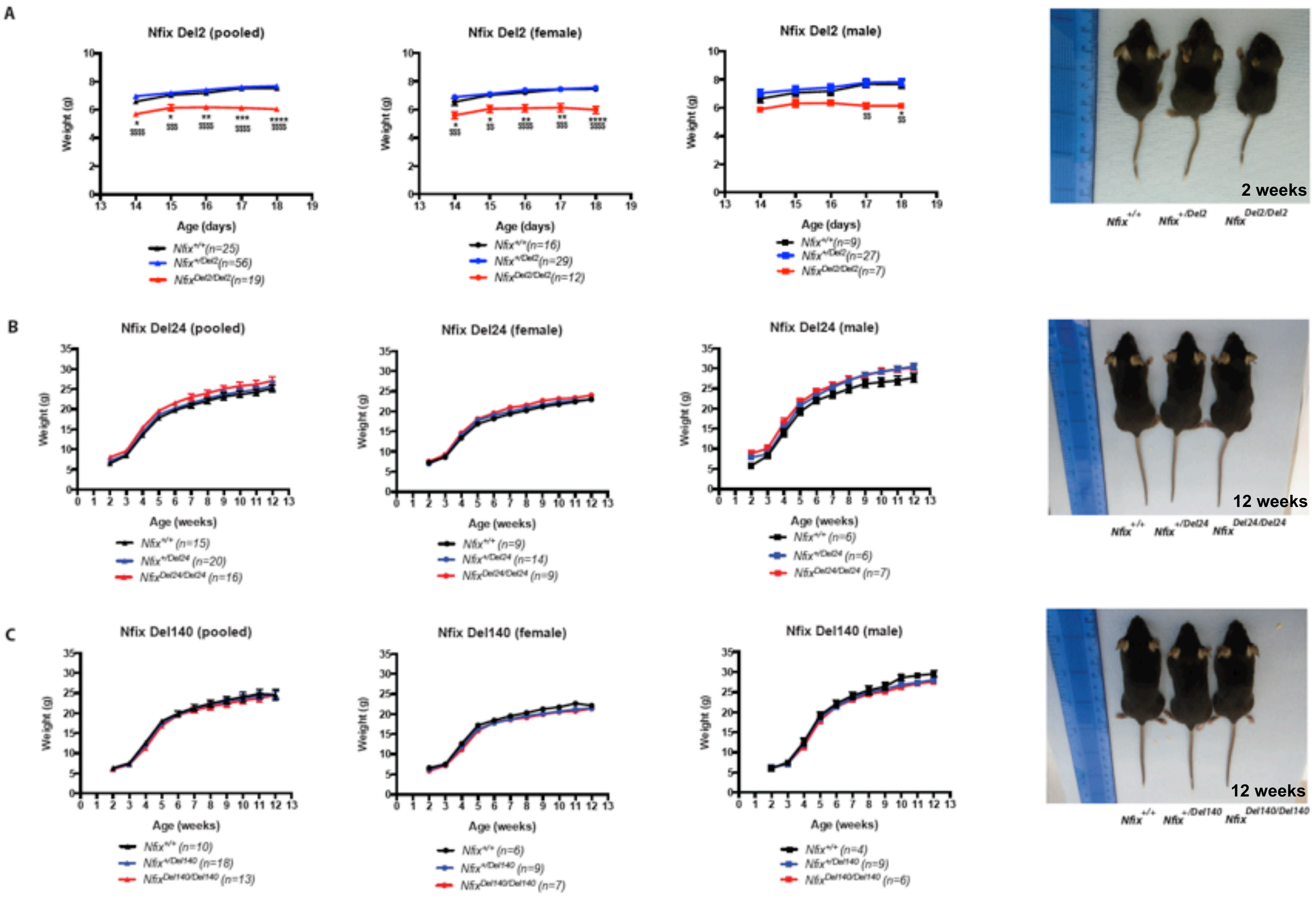
A
Nfix Del2 (pooled)
Nfix Del2 (female)
Nfix Del2 (male)
Weight (g)
Age (days)
Nfix+/+(n=25)
Nfix+/Del2(n=56)
NfixDel2/Del2(n=19)
Nfix+/+(n=16)
Nfix+/Del2(n=29)
NfixDel2/Del2(n=12)
Nfix+/+(n=9)
Nfix+/Del2(n=27)
NfixDel2/Del2(n=7)
2 weeks
Nfix+/+
Nfix+/Del2
NfixDel2/Del2
B
Nfix Del24 (pooled)
Nfix Del24 (female)
Nfix Del24 (male)
Weight (g)
Age (weeks)
Nfix+/+ (n=15)
Nfix+/Del24 (n=20)
NfixDel24/Del24 (n=16)
Nfix+/+ (n=9)
Nfix+/Del24 (n=14)
NfixDel24/Del24 (n=9)
Nfix+/+ (n=6)
Nfix+/Del24 (n=6)
NfixDel24/Del24 (n=7)
12 weeks
Nfix+/+
Nfix+/Del24
NfixDel24/Del24
C
Nfix Del140 (pooled)
Nfix Del140 (female)
Nfix Del140 (male)
Weight (g)
Age (weeks)
Nfix+/+ (n=10)
Nfix+/Del140 (n=18)
NfixDel140/Del140 (n=13)
Nfix+/+ (n=6)
Nfix+/Del140 (n=9)
NfixDel140/Del140 (n=7)
Nfix+/+ (n=4)
Nfix+/Del140 (n=9)
NfixDel140/Del140 (n=6)
12 weeks
Nfix+/+
Nfix+/Del140
NfixDel140/Del140

Supplemental Figure S5. Growth rates of *Nfix* Del2, Del24 and Del140 mice. (A) The growth curves of $Nfix^{+/+}$ (n=25), $Nfix^{+/Del2}$ (n=56) and $Nfix^{Del2/Del2}$ (n=19) mice from P14-P18 with representative images of female WT, heterozygous and homozygous *Nfix* Del2 mice at 2 weeks of age. Compared to $Nfix^{+/+}$: *p<0.05, **p<0.01, ***p<0.001, ****p<0.0001. Compared to $Nfix^{+/Del2}$: $^{\$}$p<0.05, $^{\$\$}$p<0.01, $^{\$\$\$}$p<0.001, $^{\$\$\$\$}$p<0.0001. (B) The growth curves of $Nfix^{+/+}$ (n=15), $Nfix^{+/Del24}$ (n=20) and $Nfix^{Del24/Del24}$ (n=16) mice from 2-12 weeks of age with representative images of female WT, heterozygous and homozygous *Nfix* Del24 mice at 12 weeks of age. (C) The growth curves of $Nfix^{+/+}$ (n=10), $Nfix^{+/Del140}$ (n=18) and $Nfix^{Del140/Del140}$ (n=13) mice from 2-12 weeks of age with representative images of female WT, heterozygous and homozygous *Nfix* Del140 mice at 12 weeks of age. Data are represented as mean±SEM.

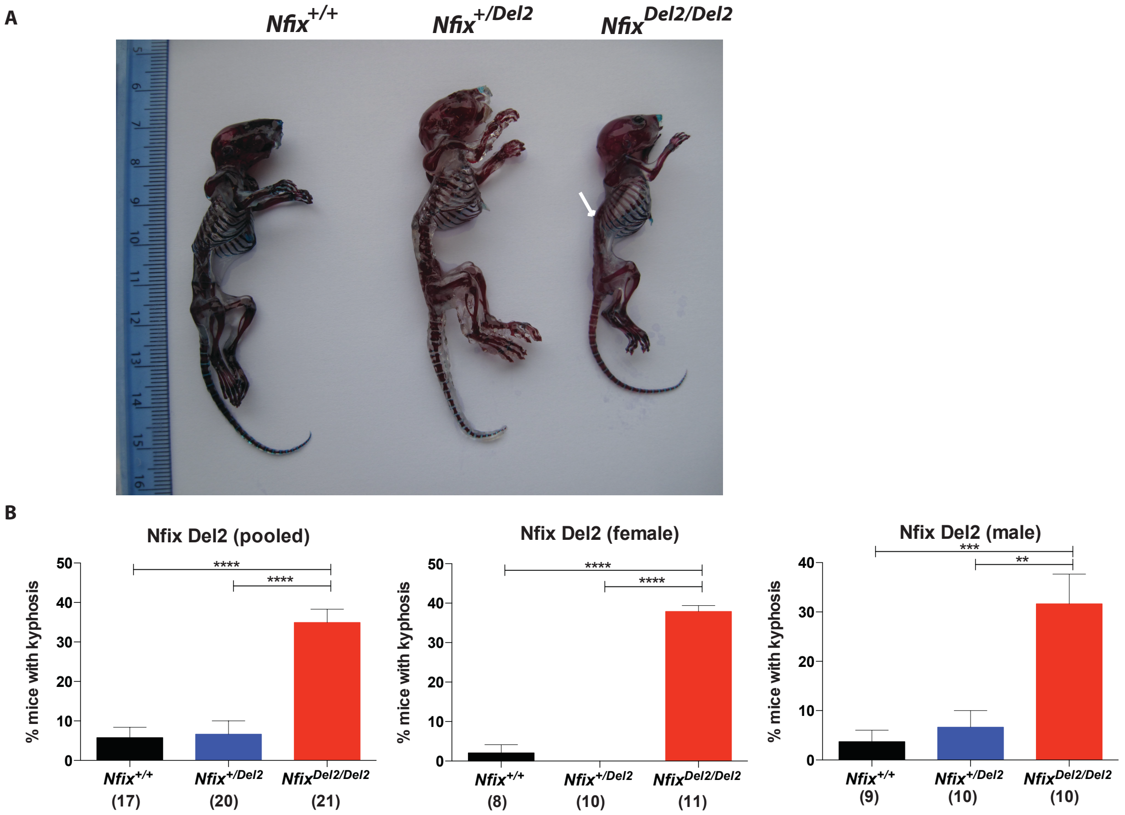

Supplemental Figure S6. Analyses of skeletal abnormalities in *Nfix* Del2 mice at 2-3 weeks of age. (A) Representative images of Alcian blue (cartilage) and Alizarin red (bone) stained skeletons of female *Nfix*$^{+/+}$, *Nfix*$^{+/Del2}$ and *Nfix*$^{Del2/Del2}$ mice. *Nfix*$^{Del2/Del2}$ mice have kyphosis (white arrow) of the thoraco-lumbar spine**.** (B) Kyphosis was assessed in 17-21 mice (indicated in parentheses) by 6 independent (blinded) observers and was found to be significantly more frequent in both male and female *Nfix*$^{Del2/Del2}$ mice.

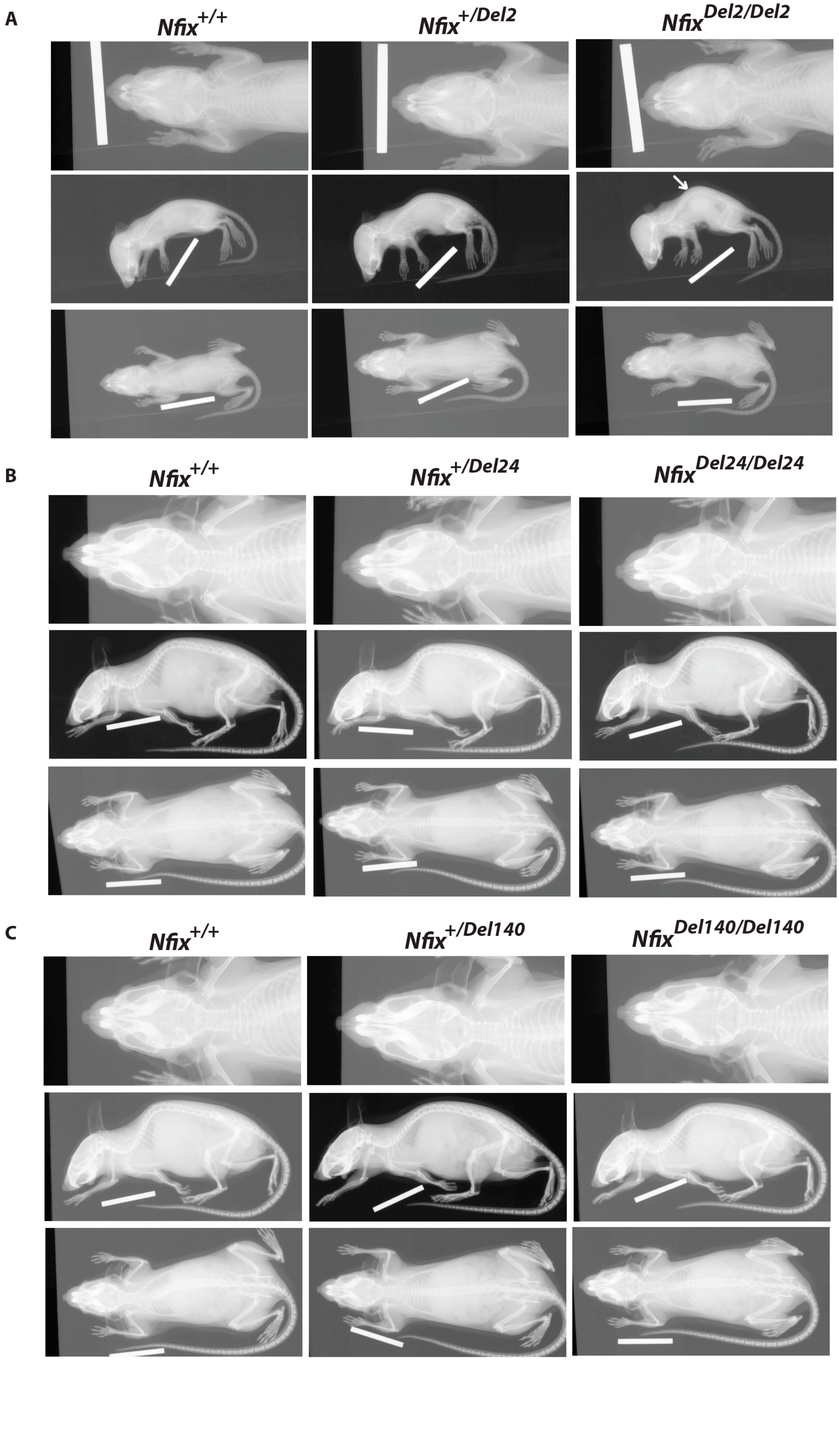
A
Nfix+/+
Nfix+/Del2
NfixDel2/Del2
B
Nfix+/+
Nfix+/Del24
NfixDel24/Del24
C
Nfix+/+
Nfix+/Del140
NfixDel140/Del140

Supplemental Figure S7. Radiological analyses of *Nfix* Del2 mice at 2-3 weeks of age and *Nfix* Del24 and *Nfix* Del140 mice at 12 weeks of age. Representative X-ray images of skulls and skeletons of female (A) *Nfix*$^{+/+}$, *Nfix*$^{+/Del2}$ and *Nfix*$^{Del2/Del2}$, (B) *Nfix*$^{+/+}$, *Nfix*$^{+/Del24}$ and *Nfix*$^{Del24/Del24}$ and (C) *Nfix*$^{+/+}$, *Nfix*$^{+/Del140}$ and *Nfix*$^{Del140/Del140}$ mice. *Nfix*$^{Del2/Del2}$ mice have kyphosis (white arrow) of the thoraco-lumbar spine**.** The scale bar represents 2 cm.

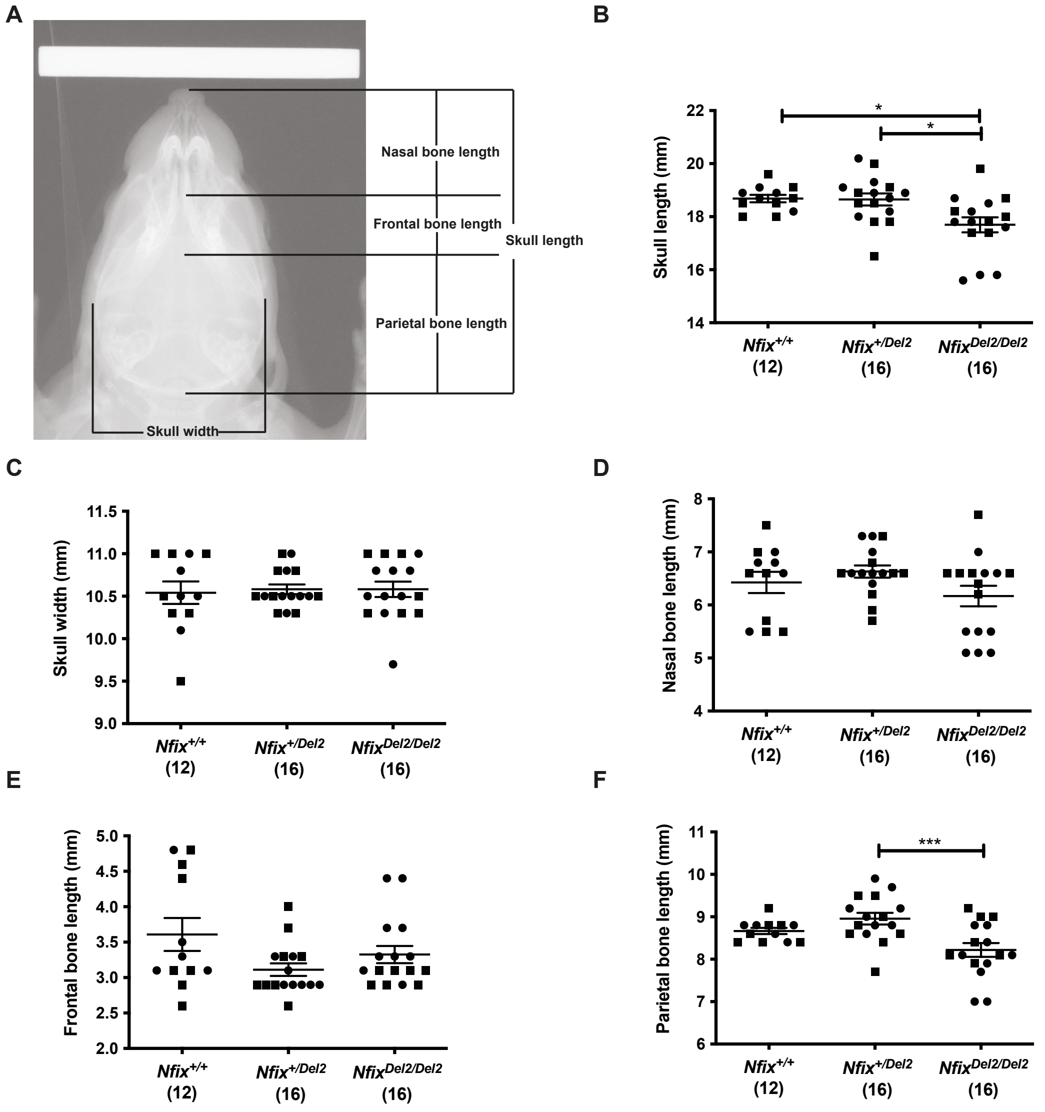

Supplemental Figure S8. Craniofacial measurements of *Nfix* Del2 mice at 2-3 weeks of age. (A) Representative X-ray image illustrating measurements of skull (B) length, (C) width, (D) nasal bone, (E) frontal bone and (F) parietal bone lengths of $Nfix^{+/+}$ (n=12), $Nfix^{+/Del2}$ (n=16) and $Nfix^{Del2/Del2}$ (n=16) mice. Data are represented as mean±SEM, scale bar represents 2 cm, *p<0.05, ***p<0.001.

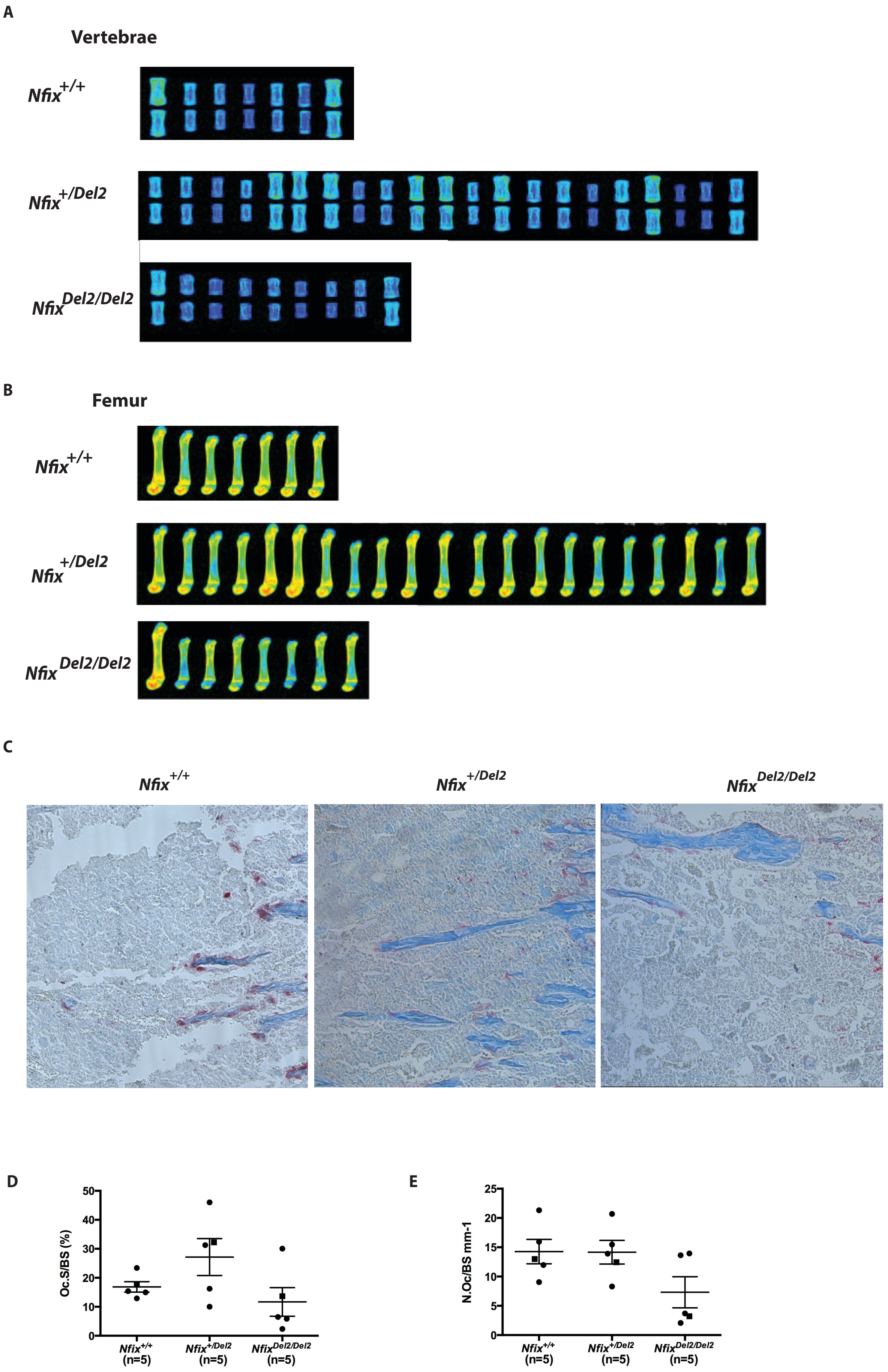
A
Vertebrae
Nfix+/+
Nfix+/Del2
NfixDel2/Del2
B
Femur
Nfix+/+
Nfix+/Del2
NfixDel2/Del2
C
Nfix+/+
Nfix+/Del2
NfixDel2/Del2
D
Oc.S/BS (%)
0
10
20
30
40
50
Nfix+/+ (n=5)
Nfix+/Del2 (n=5)
NfixDel2/Del2 (n=5)
E
N.Oc/BS mm-1
0
5
10
15
20
25
Nfix+/+ (n=5)
Nfix+/Del2 (n=5)
NfixDel2/Del2 (n=5)

Supplemental Figure S9. Characterization of skeletal phenotypes in *Nfix* Del2 mice at 2-3 weeks of age. Faxitron digital X-ray microradiographic images of (A) caudal vertebrae from $Nfix^{+/+}$ (n=7), $Nfix^{+/Del2}$ (n=21) and $Nfix^{Del2/Del2}$ (n=9) mice, as well as (B) femurs from $Nfix^{+/+}$ (n=7), $Nfix^{+/Del2}$ (n=21) and $Nfix^{Del2/Del2}$ (n=8) mice. Gray-scale images were pseudocolored according to a 16-color palette in which low mineral content is black and high mineral content is white. (C) Representative images of TRAP staining of tibias from $Nfix^{+/+}$, $Nfix^{+/Del2}$ and $Nfix^{Del2/Del2}$ mice at P21 and quantification of osteoclasts (Oc) as a (D) percentage or (E) absolute count relative to bone surface (BS). Data are represented as mean±SEM, circles represent females, squares represent males.

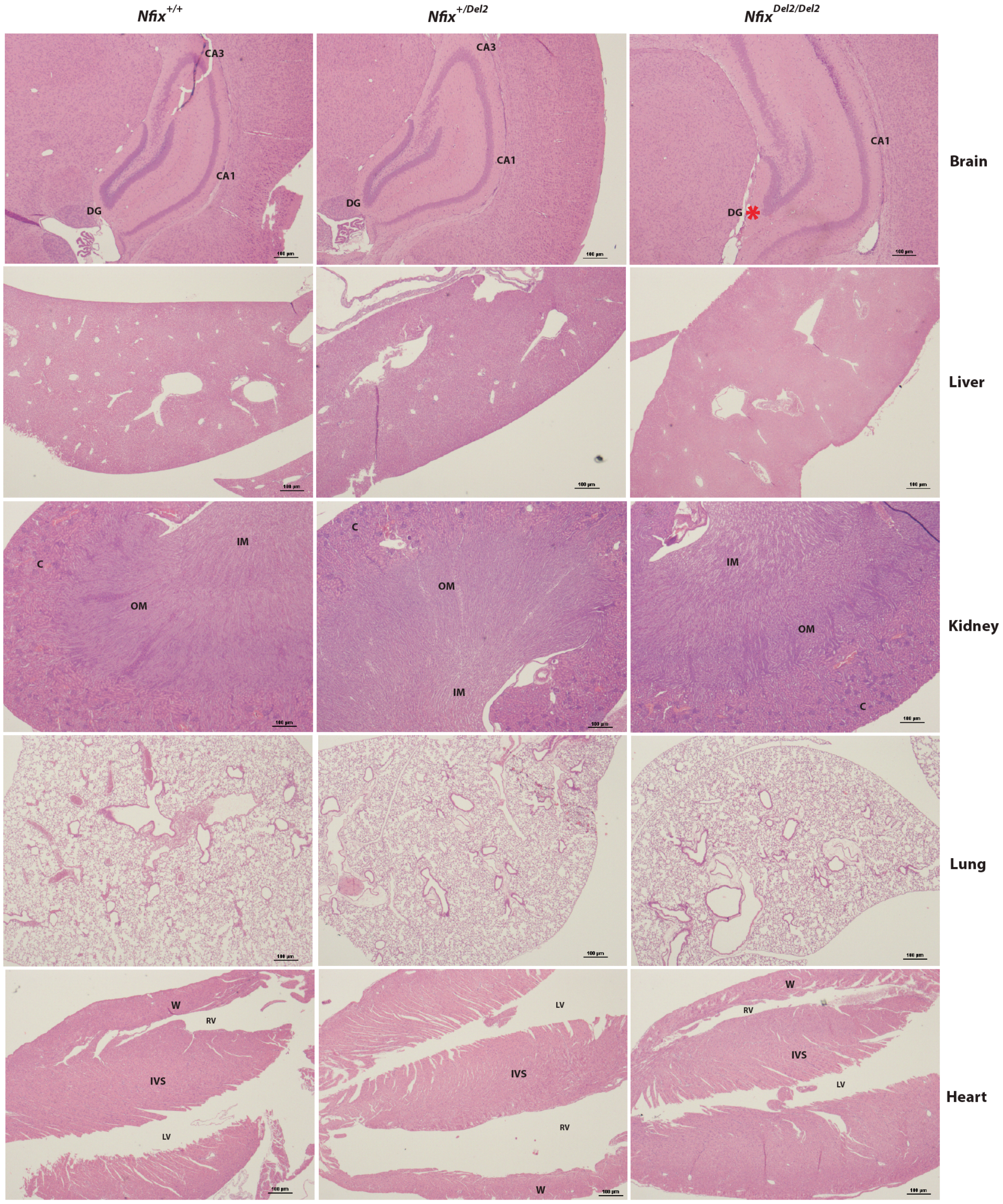

Supplemental Figure S10A. Histological analysis of brain, liver, kidney, lung and heart from *Nfix* Del2 mice at 2-3 weeks of age. Representative images of H&E staining of brain (DG: dentate gyrus; CA1 and CA3: pyramidal cell layers), liver, kidney (C: cortex; OM: outer medulla; IM: inner medulla), lung and heart (LV: left ventricle; RV: right ventricle; IVS: interventricular septum; W: wall of right ventricle) in female *Nfix*$^{+/+}$, *Nfix*$^{+/Del2}$ and *Nfix*$^{Del2/Del2}$ mice. * smaller dentate gyrus. Scale bar represents 100 µm under a 10X magnification.

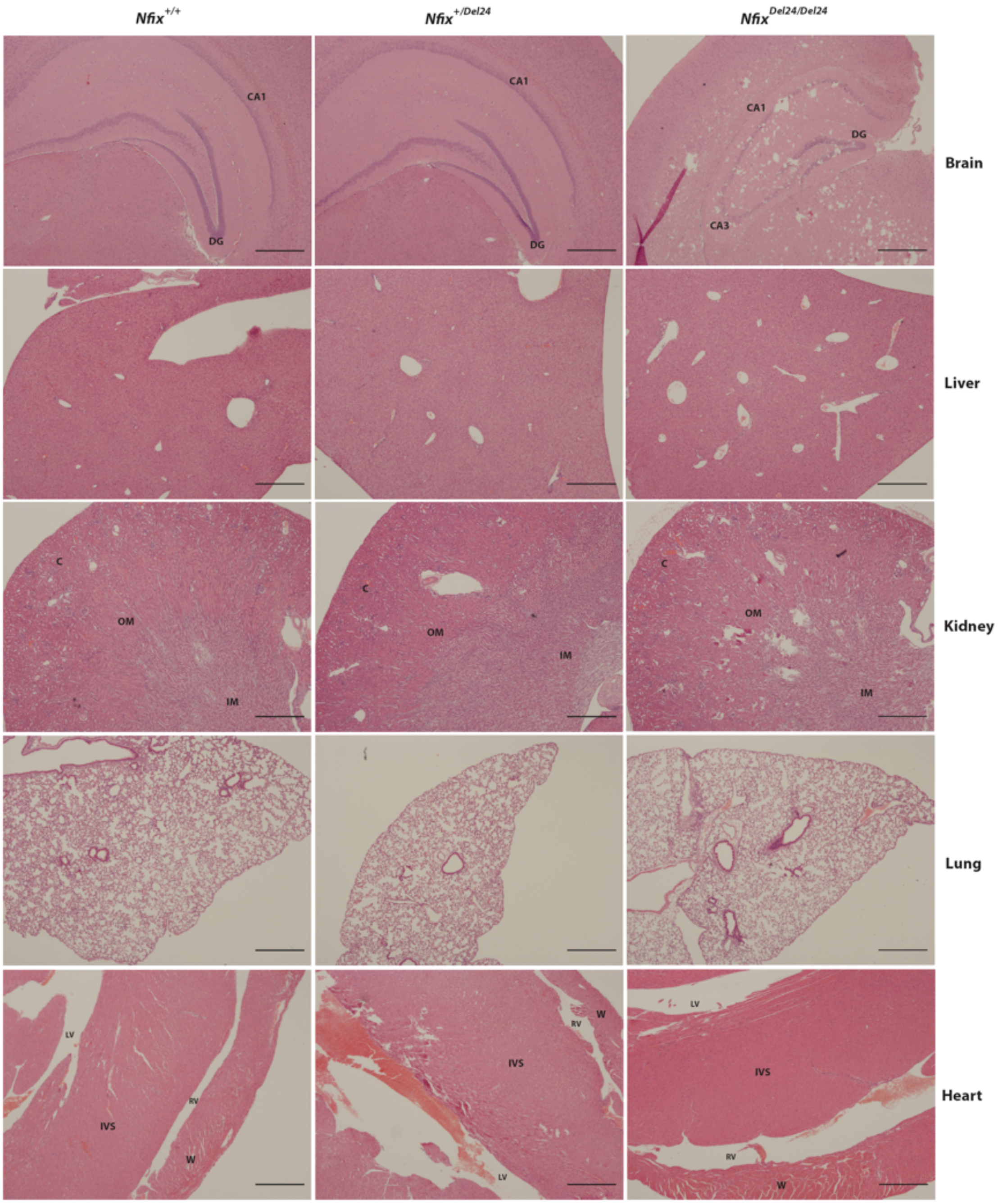

Supplemental Figure S10B. **Histological analysis of brain, liver, kidney, lung and heart from *Nfix* Del24 mice at 12 weeks of age.** Representative images of H&E staining of brain (DG: dentate gyrus; CA1 and CA3: pyramidal cell layers), liver, kidney (C: cortex; OM: outer medulla; IM: inner medulla), lung and heart (LV: left ventricle; RV: right ventricle; IVS: interventricular septum; W: wall of right ventricle) in female $Nfix^{+/+}$, $Nfix^{+/Del24}$ and $Nfix^{Del24/Del24}$ mice. Scale bar represents 100 μm under a 10X magnification.

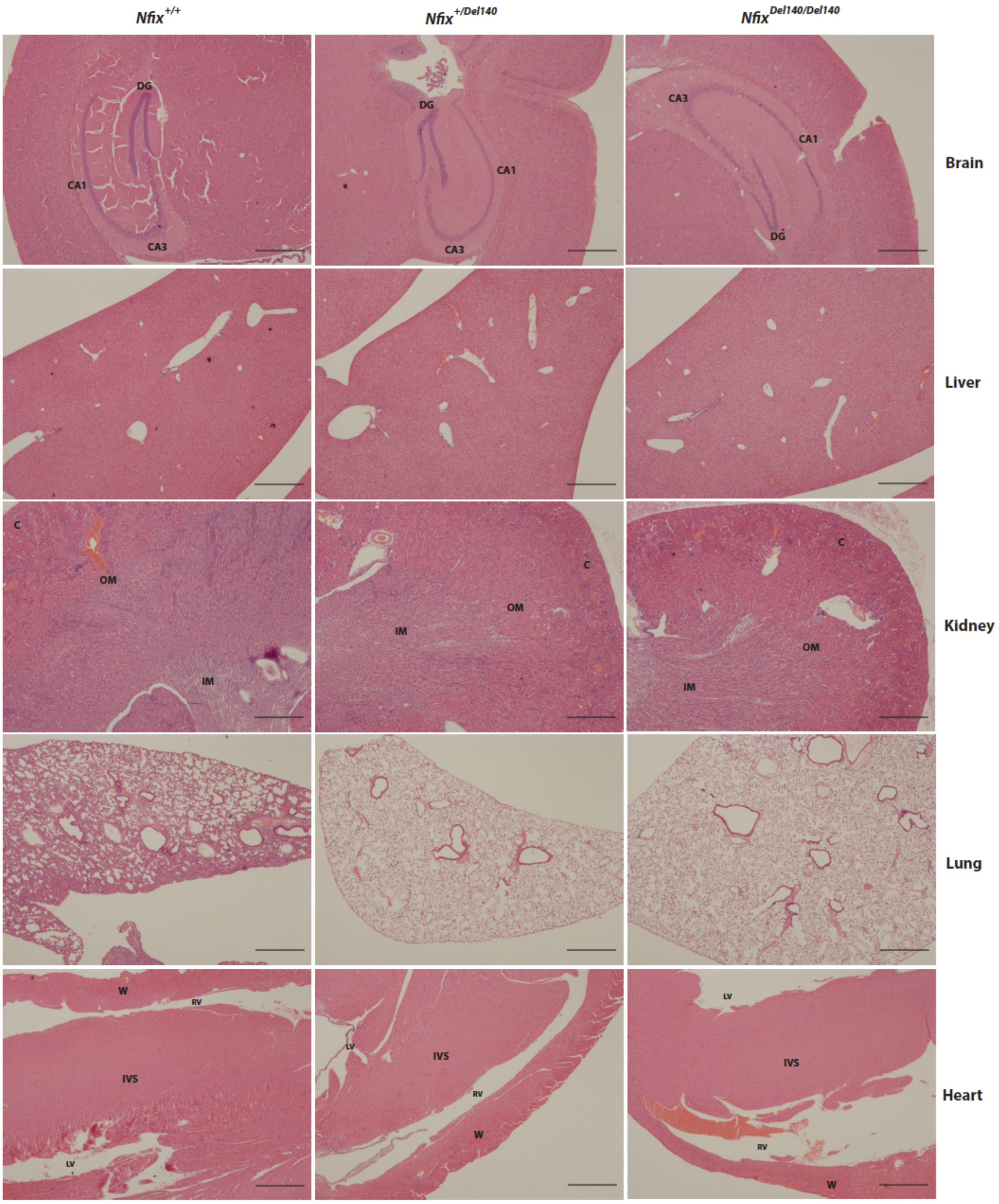

Supplemental Figure S10C. **Histological analysis of brain, liver, kidney, lung and heart from *Nfix* Del140 mice at 12 weeks of age.** Representative images of H&E staining of brain (DG: dentate gyrus; CA1 and CA3: pyramidal cell layers), liver, kidney (C: cortex; OM: outer medulla; IM: inner medulla), lung and heart (LV: left ventricle; RV: right ventricle; IVS: interventricular septum; W: wall of right ventricle) in female $Nfix^{+/+}$, $Nfix^{+/Del140}$ and $Nfix^{Del140/Del140}$ mice. Scale bar represents 100 µm under a 10X magnification.

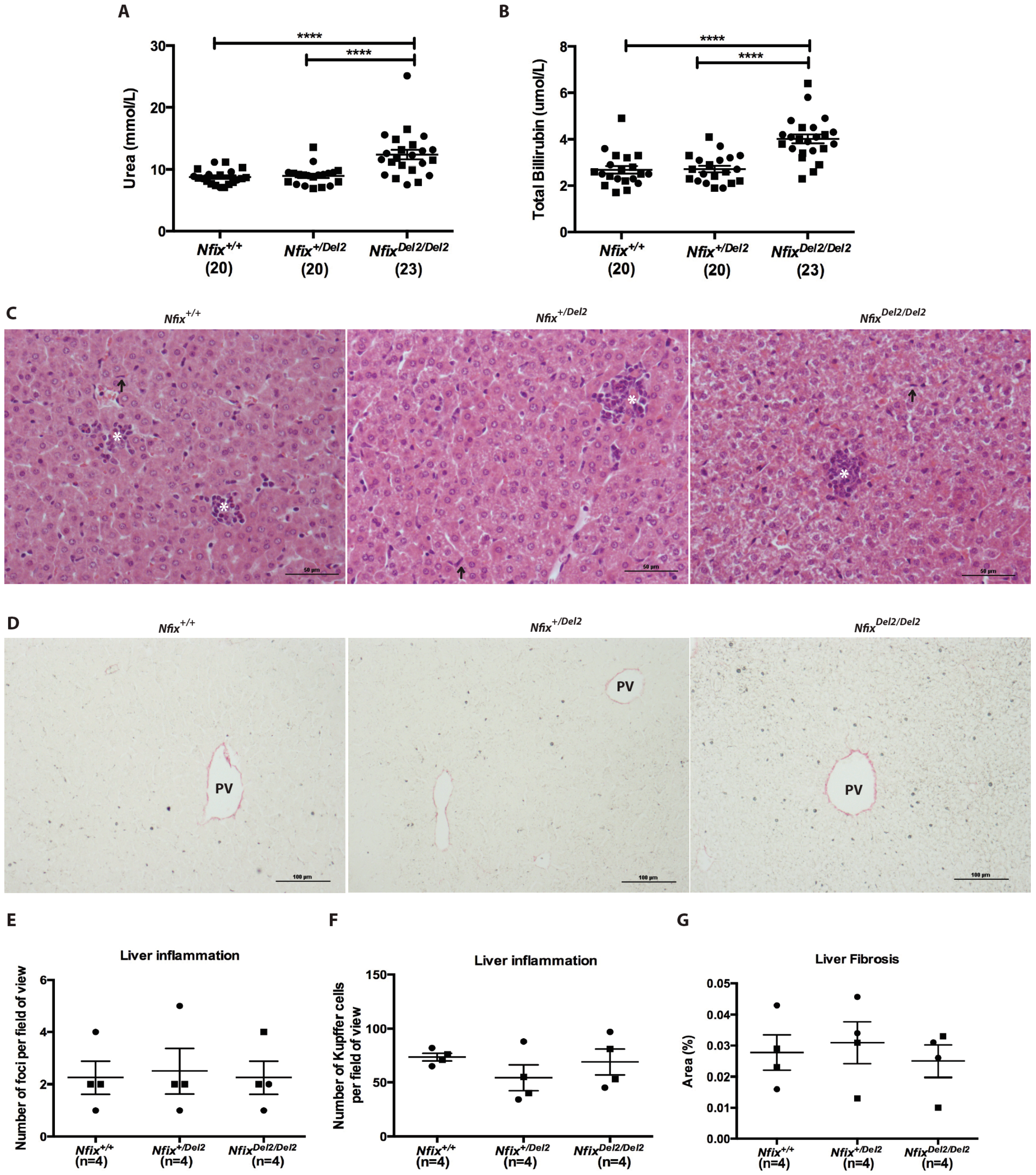

Supplemental Figure S11. Analysis of kidney and liver biochemical profiles in plasma, and histological analysis of liver in *Nfix* Del2 mice at 2-3 weeks of age. Plasma biochemistry revealed *Nfix*$^{Del2/Del2}$ mice had raised (A) urea and (B) total bilirubin compared to *Nfix*$^{+/+}$ and *Nfix*$^{+/Del2}$ mice. The number of mice analyzed is indicated in parentheses in each case. (C) Representative images of H&E stained liver sections from *Nfix*$^{+/+}$, *Nfix*$^{+/Del2}$ and *Nfix*$^{Del2/Del2}$ mice showing Kupffer cells (white asterix) as an indication of liver inflammation. (D) Sirius

Red stained livers with red stained collagenous material to identify fibrosis. The collagenous part of the portal vein (PV) also stains red. Liver inflammation and fibrosis analyzed as (E) number of foci per field of view, (F) number of Kupffer cells per field of view and (G) percentage area of fibrosis. The number of mice analyzed is indicated in parentheses in each case. **** $p<0.0001$. Data are represented as mean±SEM, circles represent females, squares represent males.

Supplemental Table S1. Comparison of features observed in MSS and MAL patients to previously reported *Nfix* deficient mouse models and our *Nfix*$^{Del2/Del2}$ mouse model. (NFA) not formally assessed; (NR) not reported; (+) present; (-) absent. The displayed phenotypes refer to recently published original and review papers(1-7).

	Patients		Mouse models		
**PHENOTYPES**	**MSS**	**MAL**	***Nfix***$^{lacZ/lacZ}$	***Nfix***$^{-/-}$	***Nfix***$^{Del2/Del2}$
**Low survival**	+	-[a]	+	+	+
**Failure to thrive**	+	-[b]	+	+	+
**Growth retardation**	+	-[c]	+	+	+
**Short stature**	+	-[d]	+	+	+
**CNS abnormalities**	+	+	+	+	+
**Skeletal abnormalities**	+	+	+	-	+
Kyphoscoliosis	+	+	+	-	+
Reduced bone mineralization	NFA	NFA	+	-	+
Osteopenia	+	NFA	-	-	+
Decreased P1NP	NFA	NFA	-	-	+
Decreased CTX	NFA	NFA	-	-	+
**Craniofacial dysmorphologies**	+	+	NR	NR	+
**Respiratory problems**	+	-	NR	NR	NFA
**Renal abnormalities**	+	NFA	NR	NR	+
**Hepatic abnormalities**	NFA	NFA	NR	NR	+
**Vision impairment**	+	+	+	+	NFA
**References**	(2,3,4,5)	(1,2,5)	(6)	(7)	This study

Unlike MSS, MAL is a disorder characterized by: [a] normal survival; [b] prenatal weight at birth > 2 standard deviations above the mean; [c] overgrowth; [d] postnatal height > 2 standard deviations above the mean in infancy and childhood.

Supplemental Table S2A. Plasma biochemical profiles and DEXA and Echo-MRI analyses of *Nfix* Del2 mice at 2-3 weeks of age. Data are represented as mean±SEM. Compared to *Nfix*$^{+/+}$: *p<0.05, **p<0.01, ***p<0.001, ****p<0.0001. Compared to *Nfix*$^{+/Del2}$: $^{\$}$p<0.05, $^{\$\$}$p<0.01, $^{\$\$\$}$p<0.001, $^{\$\$\$\$}$p<0.0001. ↑ significantly increased; ↓ significantly reduced; F-females; M-males; n numbers are shown in parentheses.

	Pooled			Female			Male		
Test	*Nfix*$^{+/+}$	*Nfix*$^{+/Del2}$	*Nfix*$^{Del2/Del2}$	*Nfix*$^{+/+}$	*Nfix*$^{+/Del2}$	*Nfix*$^{Del2/Del2}$	*Nfix*$^{+/+}$	*Nfix*$^{+/Del2}$	*Nfix*$^{Del2/Del2}$
**PLASMA BIOCHEMISTRY**									
**Kidney profile**									
Sodium (mmol/l)	141.5±0.6 (n=20)	140.5±0.5 (n=20)	141.2±0.4 (n=23)	142.0±1.0 (n=10)	140.1±0.5 (n=10)	141.0±0.4 (n=12)	141.0±0.5 (n=10)	140.8±1.0 (n=10)	141.4±0.6 (n=11)
Potassium (mmol/l)	5.2±0.2 (n=20)	5.1±0.1 (n=20)	5.6±0.2 (n=23)	5.5±0.3 (n=10)	5.4±0.2 (n=10)	5.8±0.2 (n=12)	4.9±0.2 (n=10)	4.8±0.1 (n=10)	5.4±0.2 (n=11)
Chloride (mmol/l)	106.8±0.5 (n=20)	106.0±0.4 (n=20)	108.7±0.6 (n=23)	107.9±0.8 (n=10)	106.4±0.5 (n=10)	108.8±1.0 (n=12)	105.7±0.3 (n=10)	105.6±0.7 (n=10)	108.5±0.7 (n=11)
Urea (mmol/l)	8.8±0.3 (n=20)	9.0±0.3 (n=20)	12.4±0.8 ↑****,$$$$ (n=23)	9.1±0.4 (n=10)	8.9±0.4 (n=10)	13.0±1.3 ↑*,$ (n=12)	8.5±0.3 (n=10)	9.0±0.6 (n=10)	11.8±0.8 ↑**,$$ (n=11)
Creatinine (µmol/l)	13.6±0.8 (n=20)	13.4±1.0 (n=20)	12.6±0.9 (n=23)	13.1±0.9 (n=10)	12.2±0.3 (n=10)	12.0±1.3 (n=12)	14.1±1.2 (n=10)	14.6±1.8 (n=10)	13.4±1.4 (n=11)
**Liver profile**									
ALP (U/l)	612.2±34.9 (n=20)	679.7±22.5 (n=20)	959.4±37.4 ↑****,$$$$ (n=23)	566.9±28.1 (n=10)	656.9±26.5 (n=10)	995.0±45.1 ↑****,$$$$ (n=12)	657.5±62.5 (n=10)	702.5±36.3 (n=10)	920.6±60.8 ↑**,$ (n=11)
AST (U/l)	102.4±6.9 (n=20)	86.7±5.6 (n=20)	96.4±4.9 (n=23)	103.6±8.8 (n=10)	89.3±9.7 (n=10)	96.6±6.8 (n=12)	101.2±11.1 (n=10)	84.0±6.0 (n=10)	96.2±7.3 (n=11)
Albumin (g/l)	21.7±0.5 (n=20)	21.2±0.5 (n=20)	21.7±0.5 (n=23)	21.4±0.7 (n=10)	21.8±0.7 (n=10)	21.8±0.8 (n=12)	21.9±0.7 (n=10)	20.6±0.7 (n=10)	21.5±0.8 (n=11)
Total Billirubin (µmol/l)	2.7±0.2 (n=20)	2.7±0.1 (n=20)	4.0±0.2 ↑****,$$$$ (n=23)	2.7±0.2 (n=10)	2.8±0.2 (n=10)	4.3±0.2 ↑****,$$$$ (n=12)	2.7±0.3 (n=10)	2.7±0.2 (n=10)	3.7±0.3 ↑*,$ (n=11)
**Bone profile**									
Corrected calcium (mmol/l)	2.5±0.0 (n=20)	2.5±0.0 (n=20)	2.5±0.0 (n=23)	2.5±0.0 (n=10)	2.5±0.0 (n=10)	2.5±0.0 (n=12)	2.5±0.0 (n=10)	2.5±0.0 (n=10)	2.5±0.0 (n=11)
Calcium (mmol/l)	2.5±0.0 (n=20)	2.5±0.0 (n=20)	2.5±0.0 (n=23)	2.5±0.0 (n=10)	2.5±0.0 (n=10)	2.5±0.0 (n=12)	2.5±0.0 (n=10)	2.5±0.0 (n=10)	2.5±0.0 (n=11)
Inorganic Phosphorus (mmol/l)	3.1±0.1 (n=20)	3.0±0.1 (n=20)	2.8±0.1 (n=23)	3.1±0.1 (n=10)	3.0±0.1 (n=10)	2.8±0.1 (n=12)	3.1±0.1 (n=10)	3.1±0.1 (n=10)	2.8±0.1 (n=11)
P1NP	830.6±28.8 (n=20)	861.4±37.8 (n=18)	649.3±34.6 ↓***,$$$ (n=19)	854.5±54.9 (n=9)	846.2±40.2 (n=9)	644.4±43.5↓**,$ (n=11)	811.0±28.5 (n=11)	876.5±66.2 (n=9)	655.9±59.8 ↓$ (n=8)
CTX	0.047±0.006 (n=16)	0.047±0.008 (n=14)	0.025±0.005 ↓*,$ (n=15)	0.043±0.012 (n=7)	0.036±0.010 (n=8)	0.020±0.007 (n=8)	0.050±0.006 (n=9)	0.063±0.008 (n=6)	0.029±0.006 ↓$$ (n=7)
**DEXA**									
BMD (g/cm^2)	0.03±0.00 (n=20)	0.03±0.00 (n=20)	0.02±0.00 (n=23)	0.03±0.00 (n=10)	0.03±0.00 (n=10)	0.03±0.00 (n=12)	0.03±0.00 (n=10)	0.02±0.00 (n=10)	0.02±0.00 (n=11)
BMC (g)	0.08±0.01 (n=20)	0.06±0.01 (n=20)	0.06±0.01 (n=23)	0.09±0.01 (n=10)	0.07±0.01 (n=10)	0.06±0.01 (n=12)	0.07±0.01 (n=10)	0.06±0.01 (n=10)	0.05±0.01 (n=11)
Body composition (% fat)	13.8±1.7 (n=20)	17.0±1.2 (n=20)	14.5±1.5 (n=23)	11.2±2.5 (n=10)	17.0±1.9 (n=10)	13.5±2.2 (n=12)	16.4±2.1 (n=10)	17.1±1.5 (n=10)	15.6±2.0 (n=11)
TTM (g)	7.0±0.2 (n=20)	6.9±0.2 (n=20)	5.3±0.2 ↓****,$$$$ (n=23)	7.0±0.2 (n=10)	7.0±0.2 (n=10)	5.2±0.3 ↓****,$$$$ (n=12)	7.0±0.4 (n=10)	6.8±0.3 (n=10)	5.4±0.3 ↓**,$ (n=11)
**ECHO-MRI**									
Fat (g)	0.8±0.1 (n=19)	0.7±0.0 (n=20)	0.3±0.0 ↓****,$$$$ (n=23)	0.8±0.0 (n=9)	0.8±0.1 (n=10)	0.3±0.1 ↓****,$$$$ (n=11)	0.8±0.1 (n=10)	0.7±0.1 (n=10)	0.3±0.0 ↓**,$ (n=12)
Lean (g)	6.0±0.2 (n=20)	5.7±0.1 (n=20)	4.5±0.2 ↓****,$$$$ (n=23)	6.2±0.2 (n=10)	5.8±0.1 (n=10)	4.5±0.3 ↓****,$$$ (n=12)	5.9±0.3 (n=10)	5.6±0.2 (n=10)	4.6±0.2 ↓**,$ (n=11)
Subject weight (g)	7.3±0.3 (n=20)	7.2 ±0.2 (n=20)	5.6±0.2 ↓****,$$$$ (n=23)	7.4±0.3 (n=10)	7.3±0.2 (n=10)	5.4±0.2 ↓****,$$$$ (n=12)	7.3±0.4 (n=10)	7.1±0.3 (n=10)	5.8±0.3 ↓*,$ (n=11)
Tail length (mm)	45.6±0.6 (n=41)	44.0±0.7 (n=37)	40.0±0.8 ↓****,$$$ (n=34)	46.3±0.8 (n=23)	44.8±1.0 (n=20)	40.8±1.3↓***,$ (n=18)	44.7±0.8 (n=18)	43.1±0.8 (n=17)	39.1±1.0 ↓****,$$ (n=16)

Supplemental Table S2B. Plasma biochemical profiles and DEXA and Echo-MRI analyses of *Nfix* Del24 mice at 12 weeks of age. Data are represented as mean±SEM. F-females; M-males; n numbers are shown in parentheses.

	Pooled			Female			Male		
Test	*Nfix*$^{+/+}$	*Nfix*$^{+/Del24}$	*Nfix*$^{Del24/Del24}$	*Nfix*$^{+/+}$	*Nfix*$^{+/Del24}$	*Nfix*$^{Del24/Del24}$	*Nfix*$^{+/+}$	*Nfix*$^{+/Del24}$	*Nfix*$^{Del24/Del24}$
**PLASMA BIOCHEMISTRY**									
**Kidney profile**									
Sodium (mmol/l)	145.2±0.4	144.2±0.4	145.1±0.4	144.9±0.5	143.6±0.5	145.3±0.5	145.5±0.6	144.9±0.6	145.0±0.6
	(n=16)	(n=15)	(n=16)	(n=8)	(n=8)	(n=8)	(n=8)	(n=7)	(n=8)
Potassium (mmol/l)	4.2±0.1	4.7±0.2	4.5±0.1	4.1±0.1	4.9±0.2	4.7±0.1	4.2±0.1	4.6±0.4	4.3±0.2
	(n=16)	(n=15)	(n=16)	(n=8)	(n=8)	(n=8)	(n=8)	(n=7)	(n=8)
Chloride (mmol/l)	109.1±0.5	110.1±0.5	109.2±0.3	109.3±0.7	108.8±0.3	108.9±0.4	108.9±0.7	111.7±0.4	109.5±0.5
	(n=16)	(n=15)	(n=16)	(n=8)	(n=8)	(n=8)	(n=8)	(n=7)	(n=8)
Urea (mmol/l)	11.1±0.3	11.1 (±0.6)	10.7±0.4	11.2±0.6	10.3±0.4	10.4±0.4	11.0±0.3	12.1±1.1	10.9±0.6
	(n=16)	(n=15)	(n=16)	(n=8)	(n=8)	(n=8)	(n=8)	(n=7)	(n=8)
Creatinine (µmol/l)	9.2±0.3	12.2±1.9	9.4±0.2	9.0±0.2	10.3±0.3	9.2±0.3	9.4±0.5	14.3±4.0	9.7±0.4
**Liver profile**									
ALP (U/l)	75.4±3.4	83.6±2.6	88.3±3.7	80.0±4.1	82.0±4.5	85.8±2.2	70.9±5.4	85.4±2.3	90.8±7.1
	(n=16)	(n=15)	(n=16)	(n=8)	(n=8)	(n=8)	(n=8)	(n=7)	(n=8)
AST (U/l)	47.6±4.8	42.7±2.0	45.0±1.9	43.1±2.4	42.4±3.6	46.5±2.9	52.1±9.4	43.0±1.3	43.5±2.4
	(n=16)	(n=15)	(n=16)	(n=8)	(n=8)	(n=8)	(n=8)	(n=7)	(n=8)
Albumin (g/l)	26.3±0.4	27.7±0.2	26.2±0.5	26.6±0.5	27.6±0.4	27.1±0.5	26.0±0.7	27.9±0.3	25.2±0.9
	(n=16)	(n=15)	(n=16)	(n=8)	(n=8)	(n=8)	(n=8)	(n=7)	(n=8)
Total Billirubin (µmol/l)	1.8±0.1	2.1±0.1	2.1±0.2	1.7±0.1	2.1±0.2	2.3±0.3	1.8±0.2	2.1±0.1	1.8±0.1
	(n=16)	(n=15)	(n=16)	(n=8)	(n=8)	(n=8)	(n=8)	(n=7)	(n=8)
**Bone profile**									
Corrected calcium (mmol/l)	2.4±0.0	2.3±0.0	2.3±0.0	2.3±0.0	2.3±0.0	2.3±0.0	2.4±0.0	2.3±0.0	2.3±0.0
	(n=16)	(n=15)	(n=16)	(n=8)	(n=8)	(n=8)	(n=8)	(n=7)	(n=8)
Calcium (mmol/l)	2.4±0.0	2.3±0.0	2.3±0.0	2.3±0.0	2.3±0.0	2.3±0.0	2.4±0.0	2.3±0.0	2.3±0.0
	(n=16)	(n=15)	(n=16)	(n=8)	(n=8)	(n=8)	(n=8)	(n=7)	(n=8)
Inorganic Phosphorus (mmol/l)	1.7±0.1	1.6±0.1	1.8±0.1	1.7±0.1	1.7±0.1	1.8±0.1	1.7±0.1	1.6±0.1	1.9±0.1
	(n=16)	(n=15)	(n=16)	(n=8)	(n=8)	(n=8)	(n=8)	(n=7)	(n=8)
**DEXA**									
BMD (g/cm^2)	0.06±0.00	0.06±0.00	0.06±0.00	0.06±0.00	0.06±0.00	0.06±0.00	0.06±0.00	0.06±0.00	0.06±0.00
	(n=15)	(n=15)	(n=16)	(n=8)	(n=8)	(n=8)	(n=7)	(n=7)	(n=8)
BMC (g)	0.5±0.0	0.5±0.0	0.5±0.0	0.4±0.0	0.5±0.0	0.5±0.0	0.5±0.0	0.5±0.0	0.5±0.0
	(n=15)	(n=15)	(n=16)	(n=8)	(n=8)	(n=8)	(n=7)	(n=7)	(n=8)
Body composition (% fat)	21.1±0.6	19.4±0.8	17.7±1.3	21.7±0.7	20.0±0.3	21.6±0.8	20.5±1.0	18.7±1.8	13.9±1.4
	(n=15)	(n=15)	(n=16)	(n=8)	(n=8)	(n=8)	(n=7)	(n=7)	(n=8)
TTM (g)	24.8±0.8	25.4±1.1	25.4±0.8	22.3±0.4	21.9±0.5	22.8±0.4	27.7±0.5	29.4±0.3	27.9±0.8
	(n=15)	(n=15)	(n=16)	(n=8)	(n=8)	(n=8)	(n=7)	(n=7)	(n=8)
**ECHO-MRI**									
Fat (g)	5.3±0.2	4.9±0.3	4.4±0.3	4.8±0.2	4.4±0.1	4.9±0.3	5.7±0.4	5.5±0.5	3.8±0.4
	(n=15)	(n=15)	(n=16)	(n=8)	(n=8)	(n=8)	(n=7)	(n=7)	(n=8)
Lean (g)	19.6±0.6	20.5±0.9	21.0±0.9	17.5±0.2	17.5±0.4	17.9±0.2	22.0±0.2	24.0±0.7	24.1±1.0
	(n=15)	(n=15)	(n=16)	(n=8)	(n=8)	(n=8)	(n=7)	(n=7)	(n=8)
Subject weight (g)	25.9±0.9	26.3 ±1.1	26.2±0.9	23.0±0.4	22.6±0.5	23.3±0.4	29.3±0.3	30.6±0.3	29.1±0.8
	(n=15)	(n=15)	(n=16)	(n=8)	(n=8)	(n=8)	(n=7)	(n=7)	(n=8)
Tail length (mm)	80.9±1.2	80.2±1.0	81.0±0.6	78.8±1.0	79.0±1.3	79.4±0.5	83.0±1.6	81.8±1.3	82.3±0.5
	(n=8)	(n=9)	(n=11)	(n=4)	(n=5)	(n=5)	(n=4)	(n=4)	(n=6)

Supplemental Table S2C. Plasma biochemical profiles and DEXA and Echo-MRI analyses of *Nfix* Del140 mice at 12 weeks of age. Data are represented as mean±SEM. F-females; M-males; n numbers are shown in parentheses.

	Pooled			Female			Male		
Test	*Nfix*$^{+/+}$	*Nfix*$^{+/Del140}$	*Nfix*$^{Del140/Del140}$	*Nfix*$^{+/+}$	*Nfix*$^{+/Del140}$	*Nfix*$^{Del140/Del140}$	*Nfix*$^{+/+}$	*Nfix*$^{+/Del140}$	*Nfix*$^{Del140/Del140}$
**PLASMA BIOCHEMISTRY**									
**Kidney profile**									
Sodium (mmol/l)	146.3±0.4	145.8±0.4	146.3±0.4	145.4±0.5	144.8±0.5	145.5±0.6	147.1±0.6	146.9±0.4	147.0±0.5
	(n=16)	(n=16)	(n=16)	(n=8)	(n=8)	(n=8)	(n=8)	(n=8)	(n=8)
Potassium (mmol/l)	4.4±0.1	4.6±0.1	4.6±0.1	4.2±0.1	4.6±0.1	4.6±0.1	4.5±0.1	4.5±0.1	4.5±0.1
	(n=16)	(n=16)	(n=16)	(n=8)	(n=8)	(n=8)	(n=8)	(n=8)	(n=8)
Chloride (mmol/l)	111.4±0.5	110.3±0.4	111.3±0.4	112.1±0.7	110.4±0.6	111.8±0.6	110.8±0.5	110.3±0.6	110.8±0.4
	(n=16)	(n=16)	(n=16)	(n=8)	(n=8)	(n=8)	(n=8)	(n=8)	(n=8)
Urea (mmol/l)	10.5±0.4	11.2±0.4	10.8±0.4	10.0±0.4	11.3±0.5	10.9±0.4	11.0±0.6	11.2±0.6	10.6±0.7
	(n=16)	(n=16)	(n=16)	(n=8)	(n=8)	(n=8)	(n=8)	(n=8)	(n=8)
Creatinine (µmol/l)	10.4±0.5	9.9±0.5	10.1±0.5	10.6±0.3	10.7±0.6	10.3±0.5	10.2±1.1	9.1±0.7	9.9±0.9
	(n=16)	(n=16)	(n=16)	(n=8)	(n=8)	(n=8)	(n=8)	(n=8)	(n=8)
**Liver profile**									
ALP (U/l)	119.9±3.4	113.8±3.5	132.4±5.1	128.4±4.4	125.1±2.8	147.6±5.6	111.4±3.2	102.5±2.8	117.1±3.5
	(n=16)	(n=16)	(n=16)	(n=8)	(n=8)	(n=8)	(n=8)	(n=8)	(n=8)
AST (U/l)	45.6±1.8	51.0±6.0	68.1±13.0	43.3±1.7	55.9±9.0	61.4±10.1	47.9±3.0	46.1±8.2	74.8±24.7
	(n=16)	(n=16)	(n=16)	(n=8)	(n=8)	(n=8)	(n=8)	(n=8)	(n=8)
Albumin (g/l)	27.4±0.4	27.6±0.3	27.4±0.3	28.7±0.3	28.1±0.4	28.4±0.3	26.0±0.5	27.1±0.5	26.4±0.4
	(n=16)	(n=16)	(n=16)	(n=8)	(n=8)	(n=8)	(n=8)	(n=8)	(n=8)
Total Billirubin (µmol/l)	2.2±0.1	2.2±0.2	2.5±0.2	2.2±0.1	2.4±0.3	2.4±0.2	2.2±0.2	2.0±0.3	2.5±0.4
	(n=16)	(n=16)	(n=16)	(n=8)	(n=8)	(n=8)	(n=8)	(n=8)	(n=8)
**Bone profile**									
Corrected calcium (mmol/l)	2.4±0.0	2.4±0.0	2.4±0.0	2.4±0.0	2.4±0.0	2.4±0.0	2.4±0.0	2.4±0.0	2.4±0.0
	(n=16)	(n=16)	(n=16)	(n=8)	(n=8)	(n=8)	(n=8)	(n=8)	(n=8)
Calcium (mmol/l)	2.4±0.0	2.4±0.0	2.4±0.0	2.4±0.0	2.4±0.0	2.4±0.0	2.4±0.0	2.4±0.0	2.4±0.0
	(n=16)	(n=16)	(n=16)	(n=8)	(n=8)	(n=8)	(n=8)	(n=8)	(n=8)
Inorganic Phosphorus (mmol/l)	1.9±0.1	1.9±0.1	2.0±0.1	1.7±0.1	2.0±0.1	2.0±0.1	2.0±0.1	1.8±0.1	2.0±0.1
	(n=16)	(n=16)	(n=16)	(n=8)	(n=8)	(n=8)	(n=8)	(n=8)	(n=8)
**DEXA**									
BMD (g/cm^2)	0.06±0.00	0.06±0.00	0.06±0.00	0.06±0.00	0.06±0.00	0.05±0.00	0.06±0.00	0.06±0.00	0.06±0.00
	(n=16)	(n=16)	(n=16)	(n=8)	(n=8)	(n=8)	(n=8)	(n=8)	(n=8)
BMC (g)	0.5±0.0	0.5±0.0	0.5±0.0	0.4±0.0	0.4±0.0	0.4±0.0	0.5±0.0	0.5±0.0	0.5±0.0
	(n=16)	(n=16)	(n=16)	(n=8)	(n=8)	(n=8)	(n=8)	(n=8)	(n=8)
Body composition (% fat)	17.2±0.6	17.5±0.8	17.0±0.6	17.1±0.6	18.9±1.0	18.4±0.6	17.3±1.1	16.0±1.0	15.6±0.8
	(n=16)	(n=16)	(n=16)	(n=8)	(n=8)	(n=8)	(n=8)	(n=8)	(n=8)
TTM (g)	24.7±1.0	25.2±1.0	24.3±0.9	21.1±0.9	21.9±0.5	20.8±0.3	28.2±0.5	28.6±0.8	27.7±0.4
	(n=16)	(n=16)	(n=16)	(n=8)	(n=8)	(n=8)	(n=8)	(n=8)	(n=8)
**ECHO-MRI**									
Fat (g)	4.3±0.3	4.4±0.2	4.1±0.1	3.6±0.3	4.2±0.3	3.8±0.2	4.9±0.4	4.6±0.3	4.3±0.2
	(n=16)	(n=16)	(n=16)	(n=8)	(n=8)	(n=8)	(n=8)	(n=8)	(n=8)
Lean (g)	20.4±0.8	20.9±0.9	20.2±0.9	17.5±0.7	17.8±0.3	17.0±0.2	23.3±0.4	24.0±0.7	23.4±0.5
	(n=16)	(n=16)	(n=16)	(n=8)	(n=8)	(n=8)	(n=8)	(n=8)	(n=8)
Subject weight (g)	25.7±1.0	26.3±1.0	25.2±0.9	22.3±1.0	22.9±0.4	21.9±0.4	29.0±0.6	29.6±0.7	28.5±0.5
	(n=16)	(n=16)	(n=16)	(n=8)	(n=8)	(n=8)	(n=8)	(n=8)	(n=8)
Tail length (mm)	79.8±0.9	79.9±1.1	77.8±0.7	78.8±1.3	78.3±1.5	78.0±1.1	80.6±1.2	81.2±1.3	77.7±1.1
	(n=9)	(n=9)	(n=10)	(n=4)	(n=4)	(n=4)	(n=5)	(n=5)	(n=6)

References

1. Priolo M, Schanze D, Tatton-Brown K, Mulder PA, Tenorio J, Kooblall K, et al. Further delineation of Malan syndrome. Human Mutation. 2018;39(9):1226-37.
2. Zenker M, Bunt J, Schanze I, Schanze D, Piper M, Priolo M, et al. Variants in nuclear factor I genes influence growth and development. American Journal of Medical Genetics Part C-Seminars in Medical Genetics.
3. Shaw AC, van Balkom IDC, Bauer M, Cole TRP, Delrue MA, Van Haeringen A, et al. Phenotype and Natural History in Marshall-Smith Syndrome. American Journal of Medical Genetics Part A. 2010;152A(11):2714-26.
4. van Balkom IDC, Shaw A, Vuijk PJ, Franssens M, Hoek HW, Hennekam RCM. Development and behaviour in Marshall-Smith syndrome: an exploratory study of cognition, phenotype and autism. Journal of Intellectual Disability Research. 2011;55:973-87.
5. Mulder PA, van Balkom IDC, Landlust AM, Priolo M, Menke LA, Acero IH, et al. Development, behaviour and sensory processing in Marshall-Smith syndrome and Malan syndrome: phenotype comparison in two related syndromes. Journal of Intellectual Disability Research. 2020;64(12):956-69.
6. Driller K, Pagenstecher A, Uhl M, Omran H, Berlis A, Gruender A, et al. Nuclear factor I X deficiency causes brain malformation and severe skeletal defects. Molecular and Cellular Biology. 2007;27(10):3855-67.
7. Campbell CE, Piper M, Plachez C, Yeh Y-T, Baizer JS, Osinski JM, et al. The transcription factor Nfix is essential for normal brain development. Bmc Developmental Biology. 2008;8.
