# Supplementary material for: A Mouse Model with a Frameshift Mutation in the Nuclear Factor I/X ( NFIX ) Gene Has Phenotypic Features of Marshall‐Smith Syndrome
Source: JBMR Plus. 2023 Mar 30;7(6):e10739. doi: 10.1002/jbm4.10739 (PMC10241085; doi:10.1002/jbm4.10739)
Supplement: Supplementary file 2 — Data S2. Supporting Information. [file JBM4-7-e10739-s002.pdf]

Supplemental Materials and Methods

Generation of mutant mice

One cell stage C57BL/6J embryos (The Jackson Laboratory, Maine, USA) were injected with Cas9 mRNA and guide RNA against *Nfix* exon 7 (GGTGGGTGAAAGCCATGCGTGGG). Embryos were cultured for 7 days prior to transferring to a surrogate mother. Following genotyping via PCR and Sanger sequencing, pups from the F_0 generation with mutations in exon 7 were selected to set up F_1 colonies. Pups from the F_1 population with mutations in exon 7, were backcrossed and maintained on a C57BL/6J background to breed out any potential off-target effects. Heterozygous *Nfix* mutant male and female mice were placed together to obtain litters containing wild-type, heterozygous and homozygous mice for binomial distribution analysis. All study mice were housed in a controlled environment at the MRC Harwell Institute in accordance with UK Home Office and MRC Welfare guidance. Mice were fed on a standard diet (Rat and Mouse number 3, Special Diet Services, Witham, UK) containing 1.15% calcium, 0.82% phosphate and 4088.65 units/kg vitamin D, and were provided with water ad libitum.

Genotyping analysis

Genomic DNA from mice was isolated from auricular biopsies using DNA extraction buffer (10 mM NaCl, 20 mM Tris-HCl, pH 8.0, 1 mM EDTA, 10% SDS; MilliporeSigma, Cambridge, UK) containing 0.3 mg/mL Proteinase K (Thermo Fisher Scientific, Manchester, UK) as previously described(1). Genotyping was performed by polymerase chain reaction (PCR) amplification of exon 7 using 5'-CCTACCCCAGCCAGCTAAAC-3' and 5'-TTACACTGCCCATCCCATGC-3' primers (MilliporeSigma), followed by dideoxynucleotide Sanger DNA sequencing

using the BigDye Terminator v3.1 Cycle Sequencing Kit (Life Technologies, Paisley, UK) and an ABI automated detection system (ABI 3730 Automated capillary sequencer, Applied Biosystems, Loughborough, UK). Sequences were compared with the mouse *Nfix* Ensembl reference sequence (ENSMUSG00000001911.16). PCR products were also digested with restriction endonuclease *Nla*III (New England Biolabs, Ipswich, UK) and separated by agarose gel electrophoresis. Images were acquired using a Bio-Rad Chemidoc XRS+ system UV transilluminator (Bio-Rad, Hempstead, UK).

Reverse transcription polymerase chain reaction (RT-PCR)

Murine embryonic fibroblast (MEF) cells were prepared from embryonic day 13.5 $Nfix^{+/+}$, $Nfix^{+/Del2}$, $Nfix^{Del2/Del2}$, $Nfix^{+/Del24}$, $Nfix^{Del24/Del24}$, $Nfix^{+/Del140}$ and $Nfix^{Del140/Del140}$ mice using standard protocols[(2)] and immortalized by serial passaging as previously described. Total RNA was extracted from the MEFs using the RNeasy Mini Kit (Qiagen, Manchester, UK). RNA concentration and quality were determined using a NanoDrop ND-1000 Spectrophotometer (NanoDrop Technologies, Loughborough, UK), and RNA integrity determined by agarose gel electrophoresis. Up to 1μg of total RNA was converted to cDNA using the QuantiTect Reverse Transcription Kit (Qiagen). RT-PCR reaction was performed by PCR amplification using 5'-CGACGACAGTGAGATGGAGA-3' and 5'-GGTGTGTGAAATACGGGCTC-3' primers (MilliporeSigma) in exons 6 and 8, respectively. The products were separated by agarose gel electrophoresis and visualized using a Bio-Rad Chemidoc XRS+ system UV transilluminator (Bio-Rad). The products were excised from the agarose gel, cleaned using the Qiaquik gel extraction kit (Qiagen) according to the manufacturer's instructions, followed by dideoxynucleotide Sanger DNA sequencing using the BigDye

Terminator v3.1 Cycle Sequencing Kit (Life Technologies) and an ABI 3730 automated capillary sequencer (Applied Biosystems). Sequences were compared with the mouse *Nfix* Ensembl reference sequence (ENSMUSG00000001911.16). PCR products were also digested with restriction endonuclease *Nla*III (New England Biolabs) and separated by agarose gel electrophoresis. Images were acquired using a Bio-Rad Chemidoc XRS+ system UV transilluminator (Bio-Rad).

Cell lines

MEFs were maintained in Dulbecco's Modified Eagle medium: nutrient mixture F-12 (DMEM/F-12) (Life Technologies), supplemented with 100U/ml penicillin, 100µg/ml streptomycin and 10% heat-inactivated fetal calf serum (FCS). Monkey kidney fibroblast (COS-7) cells (CRL-1651) were obtained from ATCC (LGC Standards, Middlesex, UK). COS-7 cells were maintained in Roswell Park Memorial Institute (RPMI) 1640 medium (Life Technologies), supplemented with 10% heat-inactivated fetal calf serum (FCS) and glutamine. All cells were incubated at 37°C, 5% CO_2 and 95% humidity.

***In vitro* expression assays**

Wild-type murine *Nfix* cDNA (I.M.A.G.E clone ID: 3491917; SourceBioscience, Nottingham, UK) was cloned into the N-terminal-FLAG pCMVTag2C expression vector (Promega, Southampton, UK). The 2 (*Nfix* Del2) and 24 (*Nfix* Del24) nucleotide deletions in *Nfix* exon 7 were introduced by site-directed mutagenesis using the QuikChange Lightning Site-Directed Mutagenesis kit (Agilent, Cheshire, UK), according to the manufacturer's instructions. COS-7 cells, at 50-70% confluence, were transiently transfected with 0.5µg/well of wild-type or mutant *Nfix* cDNA expression

constructs using Fugene transfection reagent (Promega). Cells were harvested 48h post transfection for qRT-PCR, Western blot and immunofluorescence analyses.

Quantitative real-time PCR (qRT-PCR)

Total RNA was extracted from cells using the RNeasy Mini Kit (Qiagen), according to the manufacturer's instructions. RNA concentration and quality were determined using a NanoDrop ND-1000 Spectrophotometer (NanoDrop Technologies), and RNA integrity determined by agarose gel electrophoresis. Up to 1µg of total RNA was converted to cDNA using the QuantiTect Reverse Transcription Kit (Qiagen). qRT-PCR reactions were performed using QuantiTect *Nfix*, *Nfia, Nfib, Nfic, Canx, Gapdh* and *Tbp1* primer assays (Qiagen) and QuantiTect SYBR Green PCR Kit (Qiagen), on a Rotor-Gene Q Cycler (Qiagen), as previously described(3). The relative expression of the target *Nfix* cDNA was normalized against the *Gapdh* and *Tbp1* mRNA controls using the Pfaffl method(4).

Western blot

Total protein was extracted from transiently transfected and untransfected COS-7 cells using 500µl of ice-cold lysis buffer (150mM NaCl, 50mM Tris (pH 8.0), 1% Triton X-100 (v/v) and 1x Protease inhibitor tablet (Roche, Hertfordshire, UK)), maintained in constant agitation at 4°C for 30 min, centrifuged for 10 min at 10,000 rpm and the supernatant collected. Protein concentration was determined using Bradford assay and protein samples were prepared in 4x Laemmli loading dye (BioRad, Hertfordshire, UK), boiled at 95°C for 5 min and resolved using precast 4-12.5% SDS-PAGE gel electrophoresis (BioRad). Samples were transferred onto PVDF membrane (PerkinElmer, Cambridge, UK), blocked in 5% milk in PBS and incubated with 1:2000

mouse anti-FLAG antibody (AB-1257; Abcam) or 1:2500 rabbit anti-CANX (AB-22595; Abcam) in 5% milk/PBS-T. Membranes were subsequently incubated with 1:2000 anti-mouse or 1:2500 anti-rabbit HRP-conjugated secondary antibodies (SC-2004; Santa Cruz Biotechnology, Dallas, USA), respectively, and visualized using ECL Western blotting substrate (BioRad) on a Chemidoc XRS+ system (BioRad) and densitometry analysis performed using Image J, as previously described(3).

Immunofluorescence analysis

Transiently transfected and untransfected COS-7 cells were fixed on coverslips using ice cold 100% methanol for 10 min and permeabilized using ice cold 0.25% Triton X-100/ phosphate-buffered saline (PBS) for 10 min prior to blocking with 1% bovine serum albumin (BSA)/ PBS-Tween (PBST) for 30 min at room temperature. The cells were incubated with 1:500 mouse anti-FLAG primary antibody (AB-1257; Abcam, Cambridge, UK) followed by 1:1000 anti-mouse Alexa Fluor 488 secondary antibody (Invitrogen, Leicester, UK). ProLong Gold Antifade reagent (Invitrogen) was used to mount the coverslips for microscopy studies (Leica microscope model DM4000B and DFC320 digital camera, Leica Microsystems, Milton Keynes, UK).

Dual luciferase reporter activity assays

The *GFAP* promoter, consisting of a 1.7 kb promoter region including the transcription start site and three NFIX binding sites, was cloned upstream of a promoter-less firefly luciferase gene (*luc*) in pGL4.12 (Promega), using methods as previously described(3,5). In addition, a reporter firefly luciferase construct in which the *GFAP* promoter was cloned in the reverse orientation (GFAPREV.pGL4.12) and the pGL4.12 empty vector were included as additional controls. COS-7 cells, at 50-70% confluence, were

transiently co-transfected with 0.5µg/well of a luciferase reporter construct together with 0.05µg/well of *Renilla* luciferase (pRL-TK) co-reporter vector as an internal control (Promega), and 0.5µg/well of either wild-type or mutant *Nfix* cDNA expression constructs, using Fugene transfection reagent (Promega), as previously reported[(3)]. After 48h, the cells were lysed and dual luciferase reporter activity determined using the Dual-Luciferase Reporter Assay System (Promega) and a Veritas Microplate Luminometer (Turner BioSystems, Southampton, UK), as previously described[(3)]. The ratio of the firefly luciferase to *Renilla* luciferase reporter activity was normalised against the activity in cells that were mock-transfected with the empty vectors.

RNA sequencing analysis

RNA sequencing analysis was used to investigate transcriptome differences in MEFs derived from the wild-type or mutant mice. Total RNA was isolated from $Nfix^{+/+}$, $Nfix^{+/Del2}$, $Nfix^{Del2/Del2}$, $Nfix^{+/Del24}$, $Nfix^{Del24/Del24}$, $Nfix^{+/Del140}$ and $Nfix^{Del140/Del140}$ MEFs using the RNeasy Mini Kit (Qiagen). RNA concentration and quality were determined using a NanoDrop ND-1000 Spectrophotometer (NanoDrop Technologies), and RNA integrity was determined using agarose gel electrophoresis. RNA sequencing was performed using the Illumina HiSeq platform at the Oxford Genomics Centre (Wellcome Trust Centre for Human Genetics, University of Oxford), and the transcriptome was analysed using Ingenuity Pathway Analysis software (Qiagen).

Plasma biochemical analysis

Blood samples were collected from the retro-orbital veins following terminal anaesthesia, and plasma was separated by centrifugation at 3000g for 5 minutes at 4℃. Plasma samples were analyzed for sodium, potassium, chloride, total calcium,

inorganic phosphate, total alkaline phosphatase activity, aspartate aminotransferase, alanine aminotransferase, albumin, total bilirubin, urea, creatinine and creatine kinase using a Beckman Coulter AU680 semi-automated clinical chemistry analyzer (Beckman Coulter, High Wycombe, UK), as described(6). Plasma total calcium was adjusted for variations in albumin concentrations using the formula: ((albumin-mean albumin) x0.02) + total calcium)(6). Procollagen type I N-terminal propeptide (P1NP) and C-terminal telopeptide (CTX) were measured by an enzyme immunoassay (EIA) (Immunodiagnostic Systems, Bolden, UK)(7).

Analysis of body composition

The body composition of nonanesthetized live mice were determined by quantitative nuclear magnetic resonance using the Echo-MRI Analyzer system (Echo Medical Systems, Houston, TX), as previously described(8).

Skeletal imaging using radiography, dual-energy X-ray absorptiometry (DEXA) and micro-CT scanning

Bone mineral content and density were assessed by whole body DEXA scanning on mice anesthetized by inhaled isoflurane using a Lunar Piximus densitometer (GE Medical Systems, Chalfont, UK) and DEXA images were analyzed using Piximus software, as reported(9). Anaesthetized mice were subjected to digital radiography at 26kV for 3 seconds using a Faxitron MX-20 digital X-ray system (Faxitron X-ray Corporation, Lincolnshire, USA) and X-ray images were processed using the DicomWorks software (http://www.dicomworks.com/)(9). For micro-CT scanning, skeletons from the mice were analyzed by a micro-CT scanner (Skyscan 1172a; Skyscan/Bruker, Kontich, Belgium) at 50 kV and 200 mA and scanned images were

reconstructed using Skyscan NRecon software and analyzed using the Skyscan CT analysis software (CT Analyzer v1.8.1.4, Skycan)[10].

Skeletal preparation and staining with Alcian blue and Alizarin red

After removal of skin, skeletons of E17.5 embryos and P1, P14, P21 and P84 mice were fixed in 95% ethanol, stained with Alcian blue (8GX, Sigma) for 24 hours, differentiated in 95% alcohol, treated with 1% potassium hydroxide for 2 days, washed overnight in running tap water, stained in aqueous Alizarin red S (Sigma) for 2 hours, washed in running tap water for 30 minutes, decolorized in 20% glycerine/1% potassium hydroxide for 1 week, and dehydrated through graded alcohols, as described[9]. Images were acquired using a Nikon D3 camera[9].

Faxitron digital X-ray microradiography.

Digital X-ray images were recorded at 10 μm pixel resolution using a Qados Faxitron MX20 variable kV point projection X-ray source and digital image system operating at 26 kV and 5× magnification (Cross Technologies Plc, Berkshire, UK). Magnifications were calibrated by imaging a digital micrometre, and bone lengths and caudal vertebrae heights were determined using ImageJ 1.41 software (http://rsb. info.nih.gov/ij/). Cortical bone thickness and diameter were determined in at least 10 locations at the middiaphysis in both anterior–posterior and medial–lateral projections. The relative mineral content of calcified tissues was determined by comparison with the following standards included in each image frame: a 1 mm thick steel plate, a 1 mm diameter spectrographically pure aluminium wire, and a 1 mm diameter polyester fiber. Sixteen-bit (2368 × 2340) DICOM images were converted to 8-bit Tiff images using ImageJ, and the histogram stretched from the polyester (gray level 0) to the steel (gray level

255) standards. Increasing gradations of mineralization density were represented in 16 equal intervals by a pseudocolor scheme for presentation of digital images[9-12].

Histology and immunohistochemistry

Dissected tissues were fixed overnight in neutral buffered 4% paraformaldehyde or in 10% formalin, decalcified in formical-4TM (Decal Chemical Corporation, New York, USA) for 3 days or 10% EDTA, pH 7.4, for 28 days before embedding in paraffin wax. Sections (5 µm cut onto Superfrost plus slides (VWR Limited, Lutterworth, UK); one section per mouse) were deparaffinized, rehydrated and stained with haematoxylin and eosin (H&E; Sigma), Alcian blue (8GX; Sigma) and van Gieson (Sigma), von Kossa (Sigma), tartrate-resistant acid phosphatase form 5b (TRAP) (Sigma) and Picro Sirius Red (Abcam) according to manufacturer's instructions, for microscopy studies (Leica microscope model DM4000B and DFC320 digital camera; Leica Microsystems)[9-12].

Immunofluorescence and immunohistochemistry analysis of brain.

Dissected mice brains were fixed in neutral buffered 4% paraformaldehyde for 2-3 days before embedding in paraffin wax. Sections (5 µm cut onto Superfrost plus slides (VWR Limited) were deparaffinized through a series of xylene and ethanol washes and antigen retrieval was performed in 10 mM sodium-citrate solution pH 6.0 at 95°C for 15 min. Sections were covered in blocking solution for 30 minutes containing 2% donkey serum (Vector Laboratories, Peterborough, UK) and 0.2% Triton-X-100 in PBS as previously described[13]. The primary antibodies were diluted in blocking solution and incubated with the sections overnight at 4°C. The primary antibodies were detected with fluorescently conjugated secondary antibodies (Alexa Fluor488 and Alexa Fluor647, raised in donkey) diluted in blocking solution for 2 h. Sections were washed

in PBS, and counterstained with DAPI before mounting on coverslips using PolyMount mounting media (Polysciences, Warrington, USA). The primary antibodies used were DCX (goat, Santa-Cruz sc-8066, 1:200); Ki67 (ms Bd Pharmingen (California, USA) 550609 1:100); GFAP (rat; Invitrogen, 13-0300, 1:800). For haematoxylin staining, sections were rehydrated in tap water and incubated in Mayer's Haematoxylin (Sigma-Aldrich) solution for two and half minutes. The sections were washed for 1 minute with tap water before being dehydrated in an ethanol-xylene series and mounted on a coverslip using DPX mounting medium (Ajax Finechem, Thermo Fischer Scientific). All sections were imaged at 20x magnification on an Olympus VS120 Slide Scanner. All measurements of cortical width and area were performed in QuPath(14). For DCX cell counts, the hippocampus region was selected and exported from QuPath to Fiji (ImageJ) for analysis.

Statistical analysis

Statistical analysis was performed using two-tailed Student's *t*-tests when comparing two groups, one-way ANOVA when comparing more than two groups, binomial distribution for viability analysis and Kolmogorov-Smirnov test for faxitron digital X-ray microradiographic analysis. The Bonferroni correction for multiple testing was applied(3).

References

1. Lemos MC, Harding B, Shalet SM, Thakker RV. A novel MEN1 intronic mutation associated with multiple endocrine neoplasia type 1. Clinical Endocrinology. 2007;66(5):709-13.

2. Garfield AS. Derivation of Primary Mouse Embryonic Fibroblast (PMEF) Cultures. Mouse Cell Culture: Methods and Protocols. 2010;633:19-27.

3. Newey PJ, Gorvin CM, Cleland SJ, Willberg CB, Bridge M, Azharuddin M, et al. Mutant Prolactin Receptor and Familial Hyperprolactinemia. New England Journal of Medicine. 2013;369(21):2012-20.

4. Pfaffl MW. A new mathematical model for relative quantification in real-time RT-PCR. Nucleic Acids Research. 2001;29(9).

5. Singh SK, Wilczynska KM, Grzybowski A, Yester J, Osrah B, Bryan L, et al. The Unique Transcriptional Activation Domain of Nuclear Factor-I-X3 Is Critical to Specifically Induce Marker Gene Expression in Astrocytes. Journal of Biological Chemistry. 2011;286(9):7315-26.

6. Piret SE, Esapa CT, Gorvin CM, Head R, Loh NY, Devuyst O, et al. A Mouse Model of Early-Onset Renal Failure Due to a Xanthine Dehydrogenase Nonsense Mutation. Plos One. 2012;7(9):10.

7. Hannan FM, Stevenson M, Bayliss AL, Stokes VJ, Stewart M, Kooblall KG, et al. Ap2s1 mutation causes hypercalcaemia in mice and impairs interaction between calcium-sensing receptor and adaptor protein-2. Human Molecular Genetics. 2021;30(10):880-92.

8. Babinsky VN, Hannan FM, Ramracheya RD, Zhang Q, Nesbit MA, Hugill A, et al. Mutant Mice With Calcium-Sensing Receptor Activation Have Hyperglycemia That Is Rectified by Calcilytic Therapy. Endocrinology. 2017;158(8):2486-502.

9. Esapa CT, Hough TA, Testori S, Head RA, Crane EA, Chan CPS, et al. A mouse model for spondyloepiphyseal dysplasia congenita with secondary osteoarthritis

due to a Col2a1 mutation. Journal of Bone and Mineral Research. 2012;27(2):413-28.

10. Esapa CT, Head RA, Jeyabalan J, Evans H, Hough TA, Cheeseman MT, et al. A Mouse with an N-Ethyl-N-Nitrosourea (ENU) Induced Trp589Arg Galnt3 Mutation Represents a Model for Hyperphosphataemic Familial Tumoural Calcinosis. Plos One. 2012;7(8):15.

11. Bassett JHD, Nordstrom K, Boyde A, Howell PGT, Kelly S, Vennstrom B, et al. Thyroid status during skeletal development determines adult bone structure and mineralization. Molecular Endocrinology. 2007;21(8):1893-904.

12. Bassett JHD, O'Shea PJ, Sriskantharajah S, Rabier B, Boyde A, Howell PGT, et al. Thyroid hormone excess rather than thyrotropin deficiency induces osteoporosis in hyperthyroidism. Molecular Endocrinology. 2007;21(5):1095-107.

13. Harris L, Zalucki O, Gobius I, McDonald H, Osinki J, Harvey TJ, et al. Transcriptional regulation of intermediate progenitor cell generation during hippocampal development. Development. 2016;143(24):4620-30.

14. Bankhead P, Loughrey MB, Fernandez JA, Dombrowski Y, McArt DG, Dunne PD, et al. QuPath: Open source software for digital pathology image analysis. Scientific Reports. 2017;7:7.
